# Wildfire-specific fine particulate matter and preterm birth: a US ECHO Cohort analysis

**DOI:** 10.1016/j.lanplh.2025.101324

**Published:** 2025-11-03

**Authors:** Allison R Sherris, Logan C Dearborn, Dana E Goin, Christine T Loftus, Adam A Szpiro, Joan A Casey, Sindana D Ilango, Jyoti Angal, Deborah H Bennett, Miatta A Buxton, Carlos A Camargo, Kecia N Carroll, Marissa L Childs, Camille Cioffi, Lisa A Croen, Dana Dabelea, Stephanie M Eick, Shohreh F Farzan, Assiamira Ferrara, Erika Garcia, Alison Gemmill, Frank Gilliland, Rima Habre, Irva Hertz-Picciotto, Alison E Hipwell, Deborah Hirtz, Margaret R Karagas, Daphne Koinis-Mitchell, Amii M Kress, Leslie D Leve, Donghai Liang, Kristen Lyall, Lacey A McCormack, Cindy T McEvoy, Hooman Mirzakhani, Rachel Morello-Frosch, Zhongzheng Niu, Thomas G O’Connor, Alicia K Peterson, Rebecca J Schmidt, Catherine J Karr, Amy M Padula, for the ECHO Cohort Consortium

**Affiliations:** Department of Environmental and Occupational Health Sciences, University of Washington, Seattle, WA, USA; Department of Environmental and Occupational Health Sciences, University of Washington, Seattle, WA, USA; Mailman School of Public Health, Columbia University, New York, NY, USA; Department of Environmental and Occupational Health Sciences, University of Washington, Seattle, WA, USA; Department of Biostatistics, University of Washington, Seattle, WA, USA; Department of Environmental and Occupational Health Sciences, University of Washington, Seattle, WA, USA; Department of Epidemiology, University of Washington, Seattle, WA, USA; Department of Pediatrics, Avera Research Institute, University of South Dakota School of Medicine, Sioux Falls, SD, USA; Department of Public Health Sciences, University of California, Davis, Davis, CA, USA; Department of Epidemiology, University of Michigan, Ann Arbor, MI, USA; Department of Emergency Medicine, Massachusetts General Hospital, Harvard Medical School, Boston, MA, USA; Pediatrics and Environmental Medicine, Icahn School of Medicine at Mount Sinai, New York City, NY, USA; Department of Environmental and Occupational Health Sciences, University of Washington, Seattle, WA, USA; Prevention Science Institute, University of Oregon, Eugene, OR, USA; Division of Research, Kaiser Permanente Northern California, Pleasanton, CA, USA; Lifecourse Epidemiology of Adiposity & Diabetes Center, Colorado School of Public Health, University of Colorado Anschutz Medical Campus, Aurora, CO, USA; Gangarosa Department of Environmental Health, Rollins School of Public Health, Emory University, Atlanta, GA, USA; Department of Population and Public Health Sciences, University of Southern California, Los Angeles, CA, USA; Division of Research, Kaiser Permanente Northern California, Pleasanton, CA, USA; Department of Population and Public Health Sciences, University of Southern California, Los Angeles, CA, USA; Department of Population, Family and Reproductive Health, Johns Hopkins University Bloomberg School of Public Health, Baltimore, MD, USA; Department of Population and Public Health Sciences, University of Southern California, Los Angeles, CA, USA; Department of Population and Public Health Sciences, University of Southern California, Los Angeles, CA, USA; Department of Public Health Sciences, University of California, Davis, Davis, CA, USA; Departments of Psychiatry, Psychology, and Clinical and Translational Science, University of Pittsburgh, Pittsburgh, PA, USA; Department of Neurological Sciences and Pediatrics, University of Vermont School of Medicine, Burlington, VT, USA; Department of Epidemiology, Geisel School of Medicine, Dartmouth College, Hanover, NH, USA; Department of Pediatrics, Brown University, Providence, RI, USA; Department of Epidemiology, Johns Hopkins University Bloomberg School of Public Health, Baltimore, MD, USA; Prevention Science Institute, University of Oregon, Eugene, OR, USA; Gangarosa Department of Environmental Health, Rollins School of Public Health, Emory University, Atlanta, GA, USA; AJ Drexel Autism Institute, Drexel University, Philadelphia, PA, USA; Avera Research Institute, Avera McKennan Hospital & University Health Center, Sioux Falls, SD, USA; Department of Pediatrics, Oregon Health & Science University, Portland, OR, USA; Channing Division of Network Medicine, Brigham and Women’s Hospital, Boston, MA, USA; Harvard Medical School, Boston, MA, USA; Department of Environmental Science, Policy and Management and School of Public Health, University of California, Berkeley, Berkeley, CA, USA; Department of Population and Public Health Sciences, University of Southern California, Los Angeles, CA, USA; Departments of Psychiatry, Neuroscience, and Obstetrics & Gynecology, University of Rochester, Rochester, NY, USA; Division of Research, Kaiser Permanente Northern California, Pleasanton, CA, USA; Department of Public Health Sciences, University of California, Davis, Davis, CA, USA; MIND Institute, University of California, Davis, Davis, CA, USA; Department of Environmental and Occupational Health Sciences, University of Washington, Seattle, WA, USA; Department of Pediatrics, University of Washington, Seattle, WA, USA; Department of Obstetrics, Gynecology & Reproductive Sciences, University of California, San Francisco, San Francisco, CA, USA

## Abstract

**Background:**

Exposure to PM_2·5_ from wildfire smoke during pregnancy has been implicated as a risk factor for preterm birth. We investigated this association in the prospective nationwide US Environmental Influences on Child Health Outcomes (ECHO) Cohort, focusing on prenatal wildfire PM_2·5_ exposure intensity, duration, and timing.

**Methods:**

In this cohort analysis, we included live singleton births recorded in the ECHO Cohort with available data on gestational age at birth and birthweight and dates of conception between Jan 1, 2006, and March 20, 2020. Census tract-level estimates of daily mean wildfire-derived PM_2·5_ for the years 2006–20 from a previous machine learning model were linked to residential address history. We calculated the mean concentration of daily wildfire PM_2·5_, days with wildfire PM_2·5_ (>0, ≥2·5, ≥5·0, and ≥10·0 μg/m^3^; termed smoke days) and consecutive smoke days (2, 3, or ≥4 days; termed smoke waves) above the prespecified concentration thresholds across pregnancy. Associations of cumlative pregnancy wildfire PM_2·5_ exposure with preterm birth (delivery before 37 weeks of gestation) were analysed by adjusted pooled logistic regression in the nationwide ECHO sample and in the US West census region. Associations between smoke days in gestational weeks 0–35 and preterm birth were evaluated by logistic regression in the national sample.

**Findings:**

We included 20 034 births from 30 ECHO Cohort study sites, with residences during pregnancy in all 48 contiguous US states and the District of Columbia. 1687 (8·4%) of the 20 034 infants were preterm. The mean daily wildfire PM_2·5_ concentration during pregnancy was 0·36 μg/m^3^ (SD 0·46), with exposure to a mean of 22·2 smoke days (SD 16·6) of any wildfire PM_2·5_ concentration (>0 μg/m^3^). Estimates of association between wildfire PM_2·5_ exposure metrics and preterm birth included the null in nationwide analyses; whereas, in the US West sample (N=5807), we estimated increased odds of preterm birth associated with mean daily wildfire PM_2·5_ (odds ratio [OR] 1·139 per 1-μg/m^3^ increase [95% CI 1·001–1·296]), exposure to smoke days with a wildfire PM_2·5_ concentration of 5·0 μg/m^3^ or greater (OR 1·018 per additional smoke day [1·003–1·032]) and 10·0 μg/m^3^ or greater (OR 1·030 [1·006–1·054]), and exposure to ≥4-day smoke waves of 5·0 μg/m^3^ or greater (OR 1·185 per additional smoke wave [1·044–1·347]) and 10·0 μg/m^3^ or greater (OR 1·232 [1·029–1·475]). At the national level, by week of gestation, associations with preterm birth were observed in mid-pregnancy for smoke days with wildfire PM_2·5_ concentrations above 0 μg/m^3^, of 2·5 μg/m^3^ or greater, and of 5·0 μg/m^3^ or greater, and in late pregnancy for smoke days of 10·0 μg/m^3^ or greater.

**Interpretation:**

In a prospective cohort, we observed increased odds of preterm birth associated with wildfire PM_2·5_ exposure in the western USA, with findings suggesting an exposure–response relationship for increasing exposure intensity and duration. Preterm birth was also associated with exposure to smoke days in mid-to-late pregnancy at the national level. For practice and policy, these findings support the need for public health interventions aimed at reducing exposure to wildfire smoke during pregnancy.

**Funding:**

ECHO Program, US National Institutes of Health Office of the Director.

## Introduction

Particulates from wildfire smoke represent a growing contribution to overall ambient PM_2·5_ in the USA.^1^ Research suggests that the toxicity of wildfire PM_2·5_ is elevated relative to that of ambient PM_2·5_ from other sources, due to differences in chemical composition, oxidative potential, and size distribution.^2,3^ Pregnant individuals and the developing fetus might be sensitive to the effects of wildfire-derived PM_2·5_ through pathways including oxidative stress, inflammatory responses, epigenetic programming, and direct effects of particles crossing the placental barrier.^4^ These biological pathways are implicated in the aetiology of adverse birth outcomes including preterm birth, in which delivery occurs before 37 weeks of gestation.^5^ Indeed, preterm birth has been associated with exposure to non-specific ambientPM_2·5_ and, more recently, to wildfire-derived PM_2·5_.^6–13^ About 10% of livebirths in the USA are preterm, which have a greater risk of adverse neonatal outcomes and respiratory and neurodevelopmental effects throughout the lifecourse.^5,14^

Key questions remain regarding the reproductive health effects of wildfire PM_2·5_ and potentially susceptible regions and subgroups. In the USA, previous studies have primarily focused on specific western states or localities.^7,8,12^ Regional differences in smoke composition, climate, housing quality, and opportunity for protective action during wildfire events might lead to differences in exposure or susceptibility to wildfire PM_2·5_.^15^ There are also well documented racial inequities in preterm birth rates in the USA, and there is some evidence for socioeconomic and racial disparities in the health effects of wildfire PM_2·5_.^16,17^ Finally, given the episodic nature of wildfires relative to other ambient sources of PM_2·5_, the role of exposure intensity, duration, and timing during pregnancy remains unclear.

In the present study, we investigated associations between wildfire-specific PM_2·5_ and preterm birth in the prospective nationwide Environmental Influences on Child Health Outcomes (ECHO) Cohort in the USA. We evaluated the role of wildfire smoke PM_2·5_ exposure intensity, duration, and timing during pregnancy, as well as potential effect modification by infant sex, race of the pregnant individual, geographical region, and neighbourhood poverty rate.

## Methods

### Study design and population

The ECHO Cohort is a longitudinal prospective study involving cohort study sites across the USA. 69 pregnancy and paediatric cohort study sites contributed harmonised data elements in the first cycle of ECHO (2016–23).^18^ In the present analysis, we included live singleton births recorded in the ECHO Cohort with the following criteria: (1) data on gestational age at birth and birthweight, (2) consent for future sharing of data including residential history, (3) at least one acceptable geocoded residence during pregnancy (that could be matched to point address, street address, or street name), (4) entire pregnancy residential history within the contiguous USA, and (5) date of conception between Jan 1, 2006, and March 20, 2020. Wildfire smoke PM_2·5_ exposure estimates were available up to Dec 31, 2020; we restricted the sample to births conceived at least 41 weeks before this date to ensure that preterm births were not preferentially included at the end of the study period and thus avoid fixed cohort bias. We excluded ECHO study sites with (1) selection on low gestational age or birthweight, (2) greater than 25% missingness of model 1 covariates described herein, or (3) fewer than 100 births meeting inclusion criteria. In secondary analyses, we restricted the sample to births in the 11 states of the contiguous US West census region (Arizona, California, Colorado, Idaho, Montana, Nevada, New Mexico, Oregon, Utah, Washington, and Wyoming; as defined by the US Census Bureau) due to higher wildfire smoke exposure in this region than in the other census regions, and to enable comparison with previous studies. Gestational age was determined by the following methods: best obstetrical consensus estimate; neonatal estimate of gestational age at delivery; obstetrical estimate from last menstrual period, first or second trimester ultrasound, or in-vitro fertilisation; administratively recorded estimated date of delivery; or caregiver or self-report (appendix p 3). Given that the unit of analysis in our study was individual births, this meant that individuals with multiple singleton births recorded in the ECHO Cohort during the study period could contribute data to the present analysis for more than one birth.

Study protocols for each cohort study site were reviewed by local institutional review boards and/or the designated single ECHO Program institutional review board; all participants provided written consent for the use of data for future ECHO Program research.

### Exposure assessment

We used data from a machine learning model of daily wildfire-specific PM_2·5_ across the contiguous USA for the years 2006–20 that has been described previously.^19^ Briefly, satellite imagery and simulated air trajectories from fires were used to identify days with wildfire-related smoke and infer daily mean wildfire PM_2·5_ concentrations at ground-based US Environmental Protection Agency (EPA) monitors. A machine learning model was then developed to predict wildfire PM_2·5_ concentrations using spatiotemporal inputs at a 10 km grid resolution and produce population-weighted census tract means. This model performed well on out-of-sample data from both EPA monitors and PurpleAir monitors (coefficient of determination, *R*^2^: 0·67–0·70) over the entire range of wildfire PM_2·5_ exposure, improving on previous models that tended to underestimate high wildfire PM_2·5_ concentrations.^19^

We retrospectively linked wildfire PM_2·5_ estimates to pregnant individuals by date and census tract of residence (ie, census tract on each day of pregnancy) to estimate daily wildfire PM_2·5_ exposure during pregnancy. Our metrics of overall wildfire smoke PM_2·5_ exposure were (1) the mean concentration of daily wildfire PM_2·5_ during the exposure period and (2) the number of smoke days, defined as days with exposure to wildfire PM_2·5_ greater than 0 μg/m^3^ (based on modelled estimates of daily mean values^19^) during the exposure period. To evaluate the role of exposure intensity, we calculated the number of smoke days when wildfire PM_2·5_ exceeded prespecified thresholds (≥2·5, ≥5·0, and ≥10·0 μg/m^3^) during the exposure period. These thresholds were selected a priori based on the distribution of wildfire PM_2·5_ concentrations on smoke days, corresponding to approximately the 50th, 75th, and 90th percentiles (appendix p 3). To evaluate the role of exposure duration, we calculated the number of smoke waves, defined as consecutive smoke days (2, 3, or ≥4 days) exceeding the specified thresholds.

### Statistical analysis

In descriptive statistics, we calculated mean daily wildfire PM_2·5_ concentrations and the number of smoke days and smoke waves from conception to delivery. We evaluated mean daily wildfire PM_2·5_, mean smoke days, and rates of preterm birth in the overall population and in different categories of demographic characteristics as potential effect modifiers. We also evaluated Pearson’s correlation coefficients between each metric of exposure, and between smoke days in different weeks of gestation.

We investigated preterm birth (delivery before 37 weeks of gestation) as the primary outcome and continuous gestational age at delivery as the secondary outcome. We used pooled logistic regression, a method applicable to interval-censored time-to-event data, to estimate conditional odds ratios (ORs) for preterm birth associated with wildfire smoke PM_2·5_ exposure.^20^ The analytical dataset included time-updated metrics of exposure by gestational week: the cumulative mean daily wildfire PM_2·5_ and the cumulative number of smoke days, defined from conception to the start of each gestational week. Given that preterm births in the study population occurred from 22–36 weeks’ gestation, we calculated these time-updated metrics from gestational week 22 through to delivery for each birth; term births (≥37 weeks’ gestation) were censored at 36 weeks. Models evaluated the outcome of preterm birth status at each gestational week, incorporating an indicator fixed effect for gestational week, ensuring that cumulative pregnancy exposure for a preterm birth was compared to cumulative exposure up to the same gestational week for births at risk for preterm birth. The estimates for all gestational weeks were pooled to yield the odds of preterm birth associated with a one-unit increase in cumulative exposure, conditional on the pregnancy continuing to the start of the previous gestational week.

All models were implemented as mixed-effect models with use of the lme4 package in R (version 4.4.0) with random intercepts for cohort study site. Associations were interpreted based on 95% CIs for effect estimates and whether these crossed the null. Site-specific multiple imputation by chained equations was used to impute missing covariate data using the mice and miceadds packages in R. Potential confounders and precision variables were identified a priori based on a hypothesised directed acyclic graph (appendix p 15).

We specified two models: model 1 (primary model) included infant sex, the pregnant individual’s age at delivery (spline with 3 degrees of freedom), self-reported race (American Indian or Alaska Native; Asian, Native Hawaiian, or Other Pacific Islander; Black; White; more than one race; or Other), self-reported Hispanic ethnicity, and residential census tract poverty rate (continuous; as a neighbourhood-level measure, defined as the percentage of all residents below the annual US federal poverty level by census tract for the census year most proximal to the year of birth), season of conception, infant birth year (spline with 4 degrees of freedom), and spatial thin plate regression splines (10 degrees of freedom) to control for geographical confounding. Model 2 (extended model) included additional adjustment for precision variables and those variables with higher missingness: parity (0, 1, or ≥2), prepregnancy BMI, any self-reported tobacco use during pregnancy, any self-reported alcohol consumption during pregnancy, method of determining gestational age, and education (high school degree or equivalent or less; some college [university] education, associate’s degree, or trade school; bachelor’s degree; or postgraduate degree). Education of the pregnant individual was measured at different stages of their child’s life at different sites (pregnancy: 73%, early childhood [ages 1 to <5 years]: 8%, middle childhood [ages 5 to <12 years]: 19%); we therefore included an interaction term between the reported education level and the life stage at which data were collected. Extended models excluded sites with greater than 50% missingness in any covariate. As a sensitivity analysis, we also fit models with the model 1 adjustments in this restricted sample that excluded sites with greater than 50% missingness. We evaluated associations for preterm birth separately in the nationwide sample and the US West census region.

To investigate the role of exposure timing, we implemented separate logistic regression models with adjustment for model 1 covariates to evaluate associations between smoke days in gestational weeks 0–35 and preterm birth in the nationwide sample.

We used mixed-effects linear regression with adjustment for model 1 covariates for the secondary outcome of gestational age at delivery in the nationwide sample, in which exposure was calculated from conception up to 32 weeks’ gestation to ensure a fixed exposure window and to include 99% of births; births occurring before 32 weeks’ gestational age at delivery (ie, extremely or very preterm; n=210) were excluded from these analyses.

For the exposure metrics of cumulative mean daily wildfire PM_2·5_ and smoke days, we explored effect modification of the primary outcome in the nationwide sample in stratified analyses and with interaction terms evaluated to a significance level of 0·05, adjusted for model 1 covariates. The analysed effect modifiers were infant sex, the four US census regions (West, Midwest, Northeast, and South), race of the pregnant individual, and census tract poverty rate tertiles. Self-reported race was included as a proxy for downstream effects of systemic and multilevel racism including disparities in exposures, outcomes, and opportunities for self-protective action during wildfire events.^15,17^

We also conducted the following sensitivity analyses of the primary outcome at the nationwide level: (1) evaluation of trimester-specific wildfire PM_2·5_ exposure instead of weekly exposure; (2) adjustment for pregnancy-average daily mean ambient temperature and daily mean ambient PM_2·5_;^21,22^ (3) use of fixed effects (instead of random intercepts) for cohort study site and adjustment for the nine US census divisions (New England, Middle Atlantic, East North Central, West North Central, South Atlantic, East South Central, West South Central, Mountain, and Pacific) to control for spatial confounding; (4) use of random intercepts and random effects for cohort study site in mixed models; and (5) complete case analysis instead of multiple imputation of missing covariate data.

### Role of the funding source

The funder of the study had no role in study design, data collection, data analysis, data interpretation, or writing of the report.

## Results

**Figure 1:**
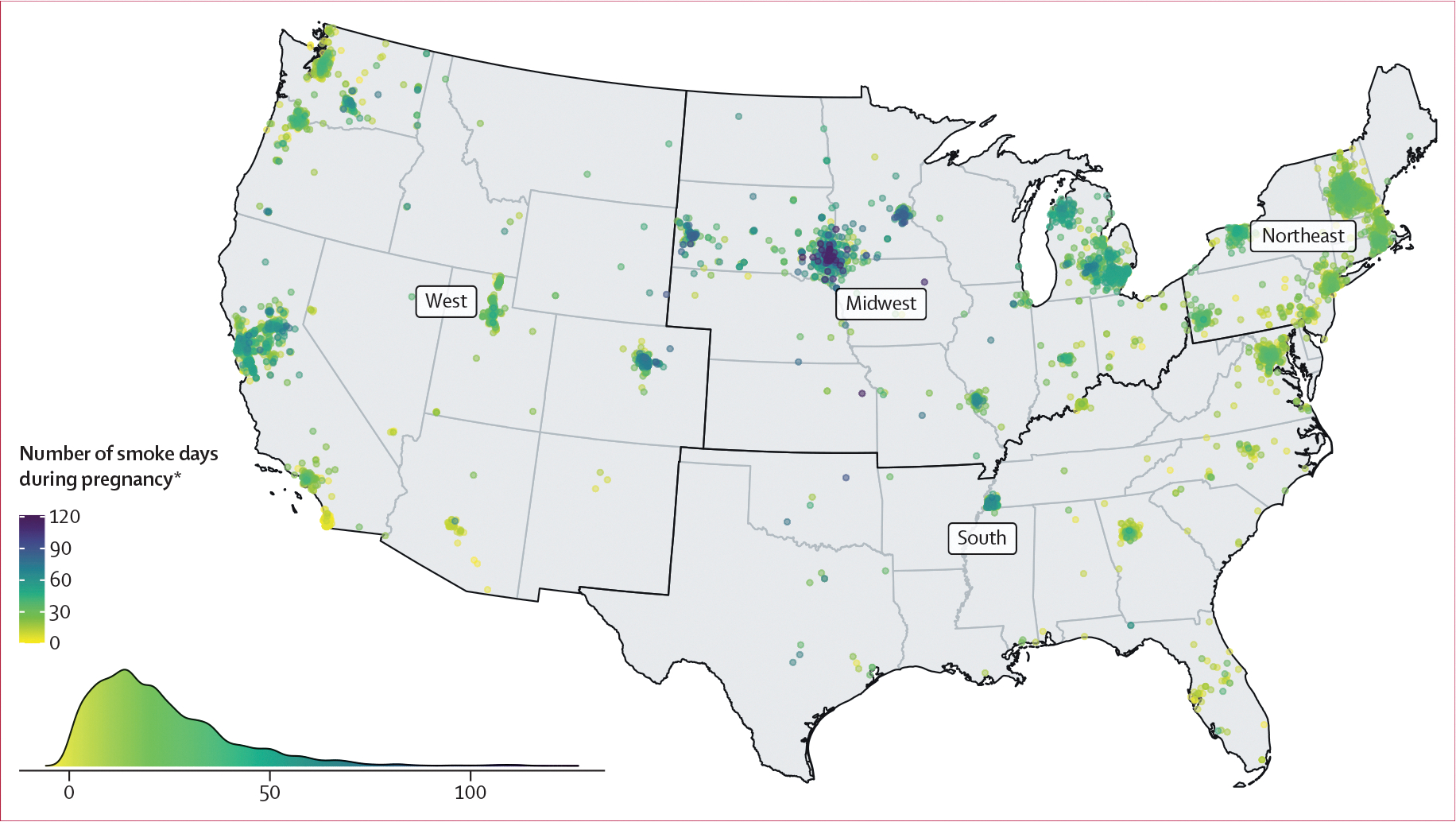
Approximate jittered locations of the first recorded residential address of pregnant individuals within the four US census regions and number of smoke days (wildfire PM_2·5_ >0 μg/m^3^) during pregnancy (N=20 034 births) *The overall mean number of smoke days was 22·2 (SD 16·6).

There were 37 371 births from 49 ECHO sites in the study period, of which 22 656 from 47 sites were singleton births with exposure and outcome data (appendix p 16). We excluded those from sites recruiting for low gestational age or birthweight (n=1490), with fewer than 100 eligible births (n=441), or with greater than 25% missingness of primary covariates (n=691), which gave a final primary study sample of 20 034 singleton births from 30 sites (appendix p 4). In this final sample, the first recorded residences during pregnancy were across all 48 contiguous US states and the District of Columbia, and thus represented all US census regions (West, n=5807 [29·0%]; Midwest, n=3570 [17·8%]; Northeast, n=6379 [31·8%]; and South, n=4278 [21·4%]; figure 1). The extended models excluded an additional 3807 births, resulting in a restricted sample of 16 227 singleton births from 18 sites. Similar to the primary sample, the first recorded residences during pregnancy in the restricted sample represented all US Census regions (47 contiguous states and the District of Columbia; appendix pp 5, 16). In the primary sample, pregnant individuals’ mean age at delivery was 30·6 years (SD 5·6; table 1). Among the 20 034 births, 12 482 (62·3%) of the pregnant individuals identified as White, 2553 (12·7%) as Black, 1342 (6·7%) as Asian, Native Hawaiian, or Other Pacific Islander, 400 (2·0%) as American Indian or Alaska Native, and 1876 (9·4%) as more than one race or Other race; 1381 (6·9%) were missing data on race. 4417 (22·0%) of the individuals identified as Hispanic.

In the primary sample, 1687 (8·4%) of the 20 034 infants were preterm, including 210 (1·0%) extremely or very preterm infants (<32 weeks’ gestation), 189 (0·9%) moderately preterm infants (32–33 weeks’ gestation), and 1288 (6·4%) late preterm infants (34–36 weeks’ gestation). The prevalence of preterm birth was higher among pregnant individuals identifying as Black (299 [11·7%] of 2553) or American Indian or Alaska Native (54 [13·5%] of 400) than among those identifying as White (953 [7·6%] of 12 482) or Asian, Native Hawaiian, or Other Pacific Islander (107 [8·0%] of 1342; table 2). Preterm infants were also slightly more likely to be in the highest tertile of neighbourhood poverty (table 2).

In most of the sample (19 872 [99·2%] of 20 034), pregnant individuals were exposed to at least one smoke day with wildfire PM_2·5_ concentration greater than 0 μg/m^3^ between conception and delivery. The mean daily wildfire PM_2·5_ concentration during pregnancy was 0·36 μg/m^3^ (SD 0·46; table 2). Pregnant individuals were exposed to a mean of 22·2 smoke days (SD 16·6) of any wildfire PM_2·5_ concentration (>0 μg/m^3^), and 1·8 smoke days (3·1) with a wildfire PM_2·5_ concentration of 10·0 μg/m^3^ or greater during pregnancy (table 2). 16 140 (80·6%) individuals were exposed to at least one smoke wave of 2 consecutive smoke days with wildfire PM_2·5_ concentration of 2·5 μg/m^3^ or greater, whereas only 1210 (6·0%) were exposed to at least one 4-day duration smoke wave of higher intensity (≥10·0 μg/m^3^; table 3). The highest mean number of smoke days during pregnancy occurred in the US Midwest region (figure 1, table 2), but the highest mean concentration of wildfire PM_2·5_ on smoke days and the highest mean number of smoke days with wildfire PM_2·5_ concentration of 10·0 μg/m^3^ or greater occurred in the US West region (appendix p 3, table 2). Mean daily wildfire PM_2·5_ exposure was higher among individuals identifying as Asian, Native Hawaiian, or Other Pacific Islander, or American Indian or Alaska Native, than among those identifying as White or Black (table 2). Wildfire PM_2·5_ exposure metrics across pregnancy were weakly to highly correlated (Pearson’s *r*: 0·31–0·92; appendix p 17). When assessing exposure to smoke days by week of gestation, exposures within a 1–3 week-period were moderately correlated (*r*: 0·41 to 0·62), while less proximal exposures were negligibly or weakly correlated (*r*: −0·13 to 0·39; appendix p 18).

**Figure 2:**
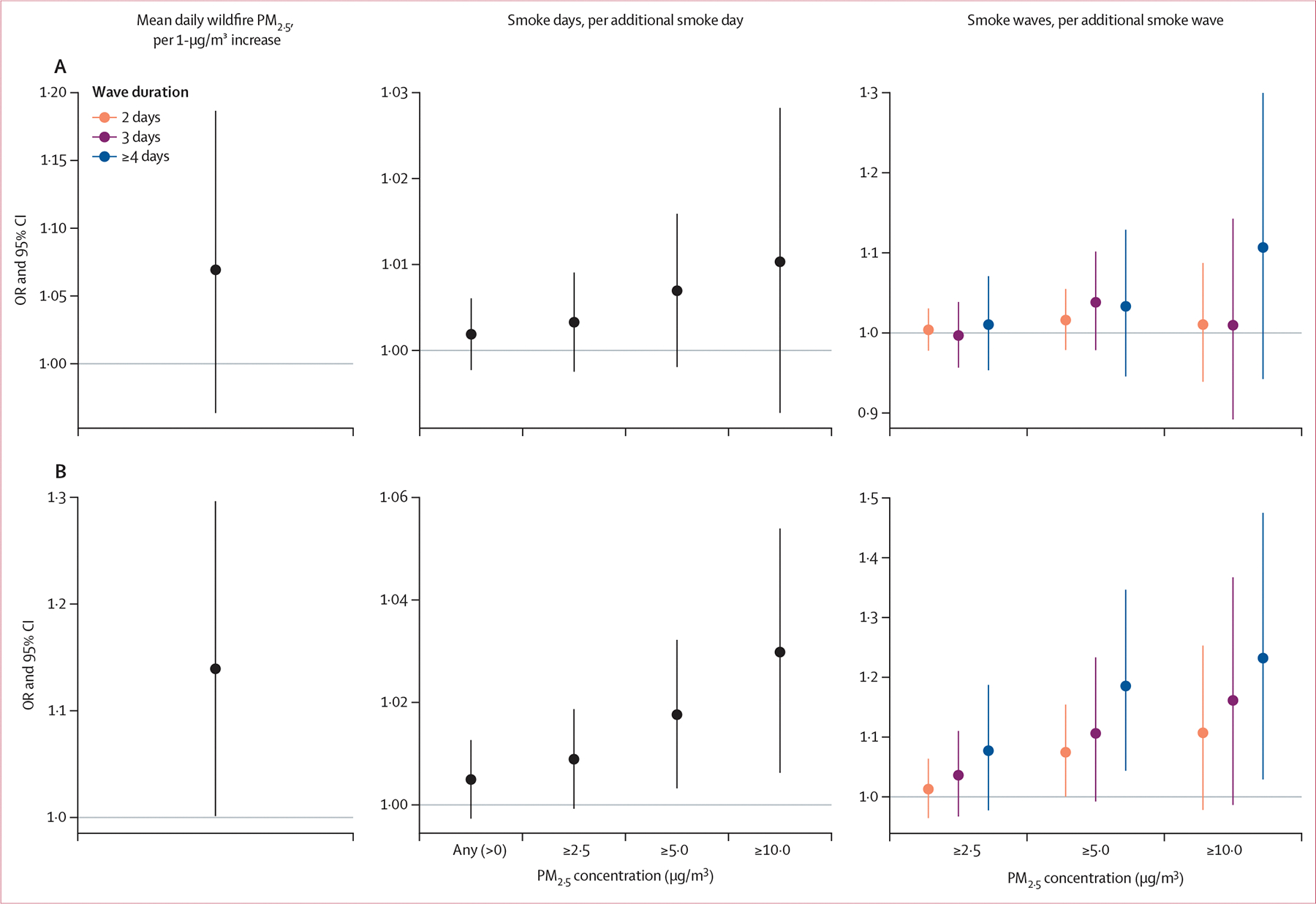
Associations between exposure to cumulative mean daily wildfire PM_2·5_, cumulative smoke days, and cumulative smoke waves during pregnancy and preterm birth in the nationwide study sample (N=20 034 births; A) and the US West study sample (N=5807 births; B) ORs are reported per 1-μg/m^3^ increase in mean daily wildfire PM_2·5_, per additional smoke day, and per additional smoke wave. Associations were analysed with pooled logistic regression adjusted for the pregnant individual’s age at delivery (spline with 3 degrees of freedom), race, and Hispanic ethnicity, infant sex, census tract (neighbourhood) poverty rate during pregnancy, season of conception, infant birth year (spline with 4 degrees of freedom), and spatial splines (10 degrees of freedom), with a random intercept for cohort study site. Note that scales on y-axes differ between plots. OR=odds ratio.

In pooled logistic regression analyses with primary model adjustment (model 1), we observed a non-significant association between cumulative mean daily wildfire PM_2·5_ concentrations in pregnancy and preterm birth, with a conditional OR of 1·069 per 1-μg/m^3^ increase (95% CI 0·964–1·187; figure 2, appendix p 6). The association between cumulative smoke days (wildfire PM_2·5_ >0 μg/m^3^) during pregnancy and preterm birth was also in the positive direction (OR 1·002 per additional smoke day [0·998–1·006]), and point estimates increased with increasing intensity of smoke days (ie, wildfire PM_2·5_ ≥2·5, ≥5·0, and ≥10·0 μg/m^3^), although the 95% CIs included the null. Associations between exposure to cumulative smoke waves and preterm birth were generally in the positive direction but not statistically significant.

In analyses restricted to the US West region (N=5807), associations between cumulative wildfire PM_2·5_ exposure metrics during pregnancy and preterm birth had larger point estimates, some of which were statistically significant, than in the nationwide analyses (figure 2, appendix p 6). There were increased odds of preterm birth associated with mean daily wildfire PM_2·5_ (OR 1·139 per 1-μg/m^3^ increase [95% CI 1·001–1·296]), exposure to smoke days with a wildfire PM_2·5_ concentration of 5·0 μg/m^3^ or greater (OR 1·018 per additional smoke day [1·003–1·032]) and 10·0 μg/m^3^ or greater (OR 1·030 [1·006–1·054]), and exposure to ≥4-day smoke waves of 5·0 μg/m^3^ or greater (OR 1·185 per additional smoke wave [1·044–1·347]) and 10·0 μg/m^3^ or greater (OR 1·232 [1·029–1·475]).

In both the nationwide and US West analyses, estimates were generally similar with extended covariate adjustment (model 2) in the restricted sample of births from sites with available covariate data (nationwide sample, N=16 227; US West sample, N=5226), when compared with model 1 estimates in the full samples (appendix p 6). However, in the US West samples, significant associations were observed for 3-day smoke waves with a wildfire PM_2·5_ concentration of 5·0 μg/m^3^ or greater and of 10·0 μg/m^3^ or greater in model 2 but not in model 1. When comparing the results of model 1 and 2 in the restricted samples, model 2 point estimates were uniformly higher but with overlapping confidence intervals compared with estimates from model 1 in both the nationwide sample and US West sample (appendix p 6). In the nationwide sample, sensitivity analyses exploring alternative assumptions yielded consistent conclusions with those from model 1 in the main analysis (appendix p 19).

For the secondary outcome, associations between exposure to wildfire PM_2·5_ from conception to 32 weeks’ gestation and gestational age at delivery were generally in the negative direction but small in magnitude, with 95% CIs spanning the null (appendix p 7).

**Figure 3:**
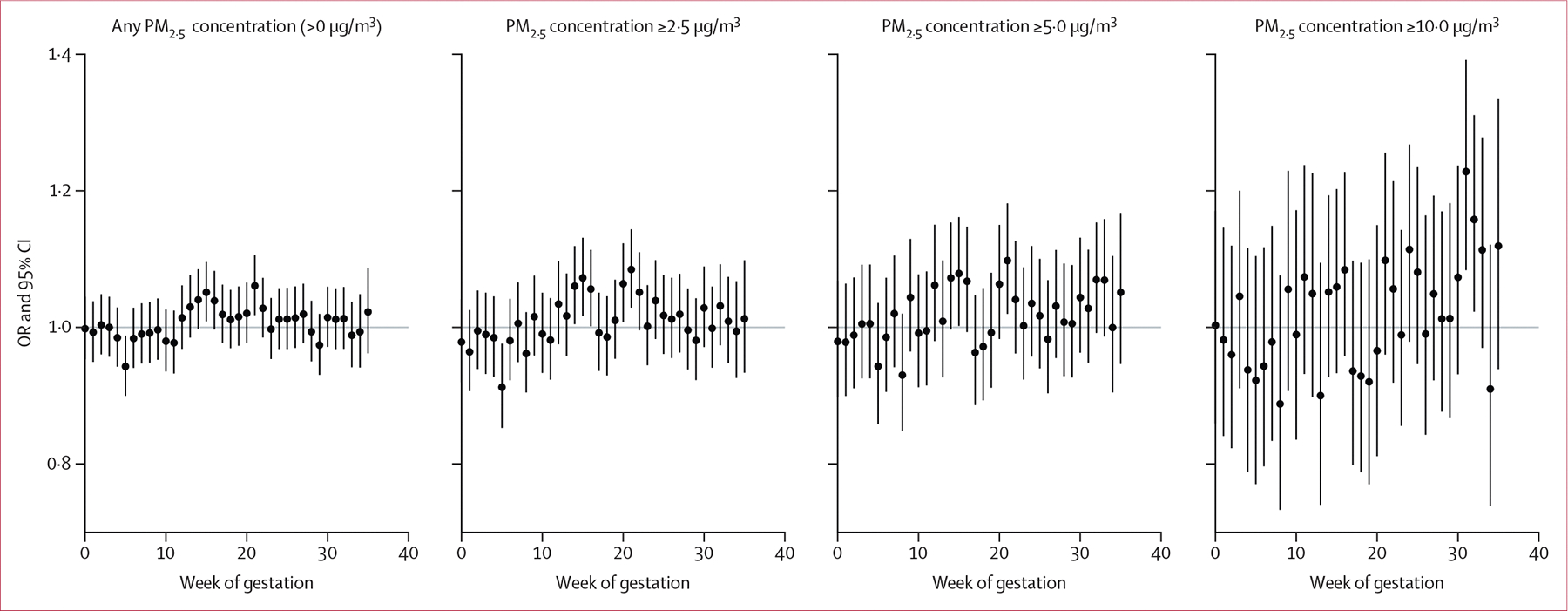
Associations between smoke days of varying intensity by week of gestation and preterm birth ORs are reported per additional smoke day. Associations were analysed with logistic regression adjusted for the pregnant individual’s age at delivery (spline with 3 degrees of freedom), race, and Hispanic ethnicity, infant sex, census tract (neighbourhood) poverty rate during pregnancy, season of conception, infant birth year (spline with 4 degrees of freedom), and spatial splines (10 degrees of freedom), with a random intercept for cohort study site. OR=odds ratio.

Evaluation of the association between smoke days by week of gestation and preterm birth identified associations in mid-pregnancy for smoke days with wildfire PM_2·5_ concentrations above 0 μg/m^3^, of 2·5 μg/m^3^ or greater, and of 5·0 μg/m^3^ or greater, with the largest effect estimates in gestational week 21 (figure 3). By contrast, smoke days with wildfire PM_2·5_ concentration of 10·0 μg/m^3^ or greater showed associations with preterm birth in late pregnancy, peaking in gestational week 31. Sensitivity analysis of trimester-specific exposures in terms of mean daily wildfire PM_2·5_, smoke days, and smoke waves consistently showed positive point estimates of association for second-trimester exposures, albeit with most 95% CIs spanning the null. There were also elevated but imprecise associations for third-trimester high-intensity exposure (appendix p 20).

**Figure 4:**
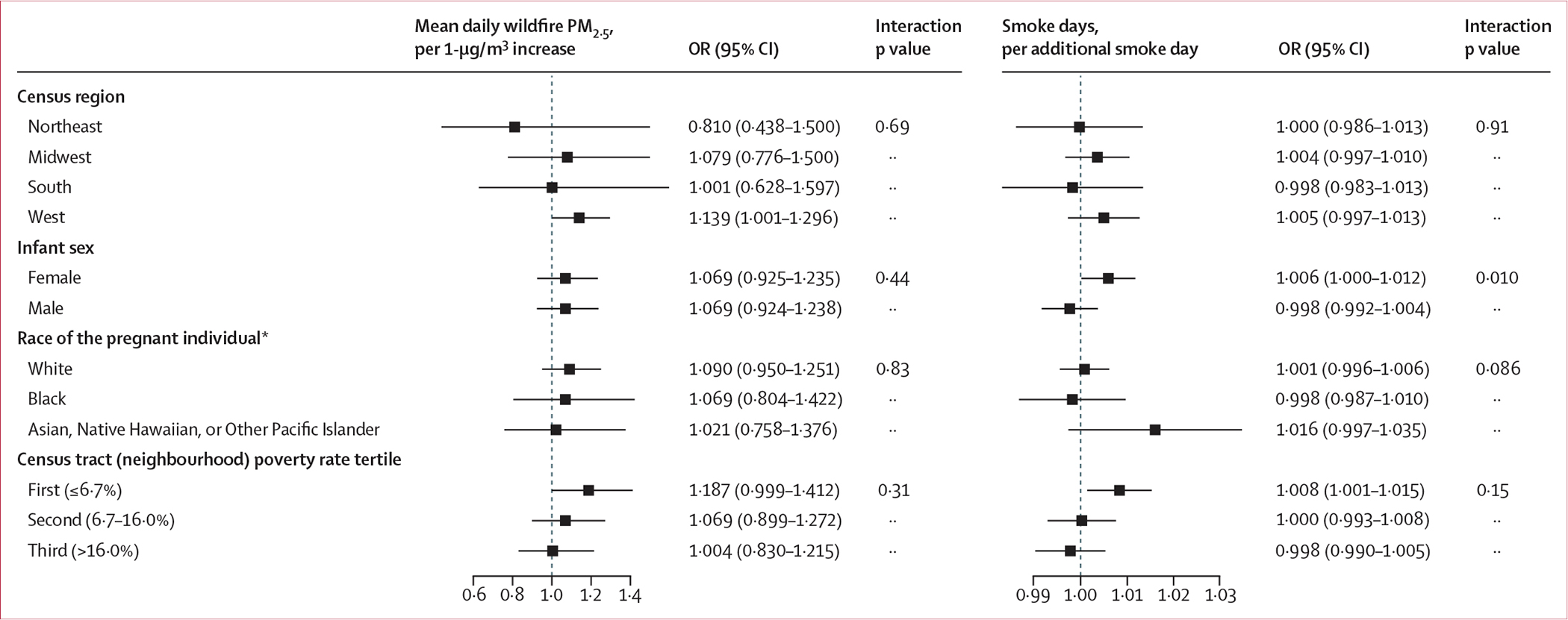
Effect modification of the relationship between cumulative wildfire PM_2·5_ exposure metrics and preterm birth ORs are reported per 1-μg/m^3^ increase in mean daily wildfire PM_2·5_ and per additional smoke day. Effect estimates were derived from stratified pooled logistic regression models. p values were obtained from multiplicative interaction terms for binary modifiers and from Wald χ^2^ tests of interaction coefficients for categorical modifiers. Note that scales on x-axes differ between plots. OR=odds ratio. *Race categories with small available sample sizes (American Indian or Native Alaskan, more than one race, and Other race) were omitted from effect modification analyses.

In effect modification analyses, point estimates of associations between cumulative wildfire PM_2·5_ exposure metrics and preterm birth were larger in the US West and Midwest regions compared with the other regions, although differences were not statistically significant (figure 4). There was a stronger association between wildfire smoke days (PM_2·5_ >0 μg/m^3^) and preterm birth among female infants relative to male infants (female infants OR 1·006 [95% CI 1·000–1·012] *vs* male infants OR 0·998 [0·992–1·004]; interaction p=0·010). There was no evidence of effect modification by race of the pregnant individual. Point estimates of association for both mean daily wildfire PM_2·5_ and smoke days were highest for births among pregnant individuals with the lowest residential poverty rates, but with interaction p values greater than 0·1.

## Discussion

We evaluated associations between exposure to wildfire-specific PM_2·5_ and preterm birth in a large, geographically diverse, well characterised prospective US cohort. In nationwide analyses, associations between cumulative pregnancy exposure to wildfire smoke PM_2·5_ and preterm birth were consistently in the hypothesised direction but imprecise and included the null. Associations were observed in mid-pregnancy for low-intensity and moderate-intensity smoke days, and in late pregnancy for high-intensity smoke days. In the US West sample, we observed increased odds of preterm birth with exposure to moderate-to-high-intensity smoke days, and with longer-duration moderate-to-high-intensity smoke waves. In all analyses, point estimates of association with preterm birth were generally larger for smoke days and smoke waves of increased intensity and duration (ie, exposure–response), and were larger with more comprehensive model adjustment in the sample of births with available covariate data. We did not identify consistent effect modification based on sex of the infant or race of the pregnant individual, although the association between smoke days and preterm birth was stronger among female infants than among male infants.

Previous studies of wildfire smoke and preterm birth have also generally identified adverse effects.^4,23^ In the USA, a 1 μg/m^3^ increase in mean daily wildfire PM_2·5_ during pregnancy was associated with increased odds of preterm birth among 534 798 births in Colorado^8^ (OR 1·055 [95% CI 1·033–1 078]) and among 5 155 026 births in California^12^ (OR 1·013 [1·008–1·017]). A study in eight southwestern US states reported elevated but null estimates of association between pregnancy mean wildfire PM_2·5_ and preterm birth.^11^ In New South Wales, Australia, an IQR increase in pregnancy mean wildfire PM_2·5_ (0·85 μg/m^3^) was associated with a hazard ratio of 1·069 (95% CI 1·058–1·081) for preterm birth among 330 884 births.^6^ These findings are similar in magnitude but more precise relative to our estimated OR of 1·069 (95% CI 0·938–1·165) for a 1-μg/m^3^ increase in mean daily wildfire PM_2·5_ with primary adjustment, and 1·080 (0·968–1·204) with extended adjustment. Heft-Neal and colleagues^7^ observed a 0·498% (95% CI 0·407–0·588) increase in the risk of preterm birth with each additional day within a wildfire smoke plume during pregnancy in California among 3 063 672 births. In the present analysis, we detected associations with smoke days only for moderate-to-high-intensity smoke days (ie, wildfire PM_2·5_ ≥5 μg/m^3^ and ≥10 μg/m^3^) in the US West. Other studies have used exposure metrics defined by the occurrence of nearby wildfires or megafires and identified associations with preterm birth.^9,10^ Differences in findings between previous studies and the present study likely stem from variable exposure assessment methods, study design, adjustment for confounding, geographical regions of focus, and outcome assessment.^4,23^

We identified larger point estimates of association between wildfire PM_2·5_ exposure and preterm birth in the US West relative to the nationwide study sample. A number of factors could contribute to this finding. First, regional differences in fuel sources and fire severity affect the composition and toxicity of wildfire-derived PM_2·5_.^24^ Second, the US West is exposed to wildfire smoke that is more recent in origin relative to other regions,^25^ and fresh wildfire smoke might have different toxicity relative to aged smoke due to physicochemical changes during long-range transport.^3^ Third, regional differences in housing characteristics, use of air conditioning, and weather conditions (including co-occurrence of wildfire smoke and heat) could potentially modify the association between wildfire PM_2·5_ and health outcomes.^16,26^ Finally, the mean concentration of wildfire PM_2·5_ on smoke days was highest in the US West, and the predictive accuracy of the exposure model was highest in the Pacific Northwest and central and northern California.^19^ Thus, effect estimates might be biased towards the null in other regions due to lower exposure heterogeneity and higher exposure measurement error.

Our findings suggest that critical windows of exposure might depend on the intensity of wildfire PM_2·5_. Models assessing smoke days by week of gestation identified associations with preterm birth in mid-pregnancy for low-to-moderate-intensity smoke days, and in late pregnancy for high-intensity smoke days. Six previous studies estimated the strongest increased risk of preterm birth with second-trimester exposure to wildfire PM_2·5_ or smoke days, with some significant associations also observed for third-trimester exposure.^6–9,11,12^ Although not the focus of the present analysis, Ha and colleagues^16^ explored wildfire smoke as a potential trigger of delivery, identifying an increased risk of delivery on days with wildfire smoke. Previous studies of ambient PM_2·5_ have also identified mid-pregnancy and near-delivery as potential windows of fetal vulnerability.^27,28^ The second trimester of pregnancy is the period of largest placental growth and angiogenesis and has been identified as a period of heightened vulnerability to the biological effects of PM_2·5_.^29^

We did not find consistent trends in effect modification across exposures and outcomes. Among female infants, the associations between wildfire smoke days of any concentration of wildfire PM_2·5_ (>0 μg/m^3^)—but not mean daily wildfire PM_2·5_—and preterm birth were stronger than among male infants. Requia and colleagues^10^ also reported stronger associations between wildfire smoke and preterm birth among female infants, while Zhang and colleagues^6^ found stronger associations between wildfire PM_2·5_ and preterm birth for male infants. We did not observe significant differences in associations between wildfire PM_2·5_ exposure metrics and preterm birth by neighbourhood poverty rate tertile or race of the pregnant individual, a finding that aligns with other studies.^7,11^

This study has limitations. First, the spatiotemporal model of wildfire PM_2·5_ has spatially heterogeneous performance, relies on US EPA monitors which are more concentrated in populated areas, and does not distinguish between wildfires, prescribed burns, and agricultural burning. However, the model incorporates spatiotemporal data inputs that have comprehensive coverage, performs well on out-of-sample data, has been validated against other recent wildfire PM_2·5_ models in California, and has been previously used in epidemiological applications.^19,30,31^ Second, we evaluated several exposure–response relationships to a significance level of 0·05, increasing the probability of incorrectly rejecting the null hypothesis. To mitigate this potential issue, we focused on the trends with respect to wildfire smoke intensity and duration when interpreting findings. Third, although the large geographical scope is a strength of our study, generally low wildfire smoke exposure in the Northeast and South census regions likely contributed to low statistical power to detect associations. We also evaluated pregnancy-average temperature as a potential confounder, but we did not explore potential interactions between temperature and wildfire PM_2·5_ exposure. Both extreme heat and extreme cold have been associated with adverse birth outcomes including preterm birth, and extreme heat might co-occur with wildfire-related PM_2·5_ exposure and have combined health effects.^16,26^ Future studies could more comprehensively explore the perinatal health effects of heatwaves in combination with wildfire PM_2·5_.

Our study has a number of notable strengths. To our knowledge, it is among the first studies to incorporate the use of smoke waves to evaluate the effect of wildfire PM_2·5_ exposure intensity and duration on adverse birth outcomes, as well as the first to use data from a longitudinal cohort rather than administrative records. We included a geographically, socioeconomically, and demographically diverse study population across the contiguous USA, building on previous literature at the state and regional level. We also had access to data on sociodemographic variables and health during pregnancy that are not typically reliable in birth records, including tobacco use and alcohol consumption during pregnancy, and prepregnancy BMI. In the sample of births with available covariate data, adjustment for these additional variables strengthened effect estimates for preterm birth. Another strength of the ECHO data is the availability of longitudinal residential history during pregnancy, rather than reliance on residence at time of delivery.

Our analysis found that the odds of preterm birth were increased with exposure to moderate-to-high-intensity smoke days and longer-duration moderate-to-high-intensity smoke waves in the US West region. Climate change and associated changes in temperature and vegetation aridity are projected to contribute to increases in the frequency, size, duration, and destructivity of wildfire activity,^32,33^ indicating the potential for more widespread population exposure to higher intensity and longer-duration wildfire episodes. Our research suggests that public health interventions to reduce exposure to wildfire smoke events could help to prevent adverse birth outcomes related to wildfire smoke exposure.

## Supplementary Material

1

## Figures and Tables

**Table 1:** Primary study population characteristics

	Preterm (N=1687)	Term (N=18 347)	Overall (N=20 034)

Infant sex			
Male	885 (52·5%)	9363 (51·0%)	10 248 (51·2%)
Female	802 (47·5%)	8977 (48·9%)	9779 (48·8%)
Missing	0	7 (<0·1%)	7 (<0·1%)
Age of the pregnant individual at delivery, years			
Mean (SD)	30·9 (6·1)	30·6 (5·5)	30·6 (5·6)
Missing	5 (0·3%)	48 (0·3%)	53 (0·3%)
Race of the pregnant individual			
White	953 (56·5%)	11 529 (62·8%)	12 482 (62·3%)
Black	299 (17·7%)	2254 (12·3%)	2553 (12·7%)
Asian, Native Hawaiian, or Other Pacific Islander	107 (6·3%)	1235 (6·7%)	1342 (6·7%)
American Indian or Alaska Native	54 (3·2%)	346 (1·9%)	400 (2·0%)
More than one race or Other race	151 (9·0%)	1725 (9·4%)	1876 (9·4%)
Missing	123 (7·3%)	1258 (6·9%)	1381 (6·9%)
Ethnicity of the pregnant individual			
Hispanic	392 (23·2%)	4025 (21·9%)	4417 (22·0%)
Non-Hispanic	1263 (74·9%)	13 928 (75·9%)	15 191 (75·8%)
Missing	32 (1·9%)	394 (2·1%)	426 (2·1%)
Education level of the pregnant individual			
High school degree or equivalent or less	429 (25·4%)	3943 (21·5%)	4372 (21·8%)
Some college (university) education, associate’s degree, or trade school	388 (23·0%)	3472 (18·9%)	3860 (19·3%)
Bachelor’s degree	349 (20·7%)	4394 (23·9%)	4743 (23·7%)
Postgraduate degree	296 (17·5%)	4018 (21·9%)	4314 (21·5%)
Missing	225 (13·3%)	2520 (13·7%)	2745 (13·7%)
Parity			
1	555 (32·9%)	6412 (34·9%)	6967 (34·8%)
2	451 (26·7%)	5613 (30·6%)	6064 (30·3%)
≥3	412 (24·4%)	3739 (20·4%)	4151 (20·7%)
Missing	269 (15·9%)	2583 (14·1%)	2852 (14·2%)
Prepregnancy BMI*, kg/m^2^			
Mean (SD)	27·6 (7·1)	26·7 (6·6)	26·8 (6·6)
Missing	228 (13·5%)	2213 (12·1%)	2441 (12·2%)
Tobacco use during pregnancy†			
Yes	148 (8·8%)	1270 (6·9%)	1418 (7·1%)
No	1326 (78·6%)	14 847 (80·9%)	16 173 (80·7%)
Missing	213 (12·6%)	2230 (12·2%)	2443 (12·2%)
Alcohol consumption during pregnancy‡			
Yes	248 (14·7%)	2985 (16·3%)	3233 (16·1%)
No	1105 (65·5%)	12 012 (65·5%)	13 117 (65·5%)
Missing	334 (19·8%)	3350 (18·3%)	3684 (18·4%)
Census region			
Midwest	336 (19·9%)	3234 (17·6%)	3570 (17·8%)
Northeast	504 (29·9%)	5875 (32·0%)	6379 (31·8%)
South	351 (20·8%)	3927 (21·4%)	4278 (21·4%)
West	496 (29·4%)	5311 (28·9%)	5807 (29·0%)
Census tract (neighbourhood) poverty rate, %			
Mean (SD)	16·2% (13·7)	14·6% (12·7)	14·7% (12·8)
Missing	12 (0·7%)	93 (0·5%)	105 (0·5%)
Season of conception			
Winter (January–March)	421 (25·0%)	4487 (24·5%)	4908 (24·5%)
Spring (April–June)	422 (25·0%)	4271 (23·3%)	4693 (23·4%)
Summer (July–September)	408 (24·2%)	4688 (25·6%)	5096 (25·4%)
Autumn (October–December)	436 (25·8%)	4901 (26·7%)	5337 (26·6%)
Infant birth year			
2006–09	148 (8·8%)	1421 (7·7%)	1569 (7·8%)
2010–13	557 (33·0%)	5554 (30·3%)	6111 (30·5%)
2014–17	585 (34·7%)	6514 (35·5%)	7099 (35·4%)
2018–21	397 (23·5%)	4858 (26·5%)	5255 (26·2%)

Data are number of singleton births (%) unless otherwise stated.

* Prepregnancy BMI was determined from recorded or self-reported measures collected between 12 months before conception through to the end of the first trimester, with observations closest to conception as the preferred measure.

† Tobacco use was defined by self-reported use of any tobacco or nicotine products, medical record abstraction, or toxicology screen (positive for nicotine or cotinine) during the ECHO pregnancy.

‡ Alcohol consumption was defined as self-reported consumption of any alcoholic beverage during the ECHO pregnancy.

**Table 2:** Proportion of preterm births and wildfire smoke PM_2·5_ exposure metrics during pregnancy in the study population by sociodemographic variables

	Study population, n	Preterm birth rate, n (%)	Mean daily wildfire PM_2·5_ (μg/m^3^)	Mean number of smoke days			
				Any (wildfire PM_2·5_ >0 μg/m^3^)	Wildfire PM_2·5_ ≥2·5 μg/m^3^	Wildfire PM_2·5_ ≥5·0 μg/m^3^	Wildfire PM_2·5_ ≥10·0 μg/m^3^

Overall	20 034	1687 (8·4%)	0·36 (0·46)	22·2 (16·6)	12·2 (10·7)	6·2 (6·3)	1·8 (3·1)
Census region							
West	5807	496 (8·5%)	0·47 (0·75)	22·2 (16·3)	13·0 (12·0)	6·0 (7·6)	2·4 (4·5)
Midwest	3570	336 (9·4%)	0·50 (0·34)	38·7 (20·9)	20·2 (13·9)	9·4 (7·8)	1·7 (2·2)
South	4278	351 (8·2%)	0·26 (0·23)	14·9 (9·9)	9·8 (7·8)	5·5 (5·1)	1·5 (2·3)
Northeast	6379	504 (7·9%)	0·24 (0·16)	18·0 (10·2)	8·6 (5·3)	5·0 (3·7)	1·4 (2·2)
Infant sex*							
Male	10 248	885 (8·6%)	0·36 (0·47)	22·3 (16·6)	12·2 (10·8)	6·2 (6·4)	1·8 (3·1)
Female	9779	802 (8·2%)	0·36 (0·46)	22·2 (16·5)	12·1 (10·6)	6·1 (6·3)	1·7 (3·0)
Race of the pregnant individual*							
White	12 482	953 (7·6%)	0·36 (0·44)	23·1 (17·5)	12·5 (11·1)	6·2 (6·4)	1·6 (2·9)
Black	2553	299 (11·7%)	0·35 (0·42)	21·1 (14·8)	12·3 (10·0)	6·6 (6·1)	1·9 (2·9)
Asian, Native Hawaiian, or Other Pacific Islander	1342	107 (8·0%)	0·45 (0·74)	21·2 (14·7)	12·1 (10·9)	6·0 (6·8)	2·4 (4·3)
American Indian or Alaska Native	400	54 (13·5%)	0·48 (0·45)	34·3 (21·0)	17·2 (14·2)	7·7 (9·3)	2·0 (3·1)
More than one race or Other race	1876	151 (8·0%)	0·32 (0·44)	20·1 (13·4)	10·4 (8·8)	5·5 (5·7)	1·9 (3·3)
Ethnicity of the pregnant individual*							
Hispanic	4417	392 (8·9%)	0·31 (0·44)	18·5 (12·9)	10·1 (8·7)	5·2 (5·5)	1·8 (3·1)
Non-Hispanic	15 191	1263 (8·3%)	0·37 (0·47)	23·4 (17·4)	12·8 (11·2)	6·4 (6·6)	1·8 (3·1)
Census tract (neighbourhood) poverty rate tertile*							
First (≤6·7%)	6643	493 (7·4%)	0·37 (0·46)	22·4 (17·8)	12·7 (11·4)	6·4 (6·4)	1·7 (3·0)
Second (6·7–16·0%)	6643	558 (8·4%)	0·37 (0·50)	22·7 (16·5)	12·3 (10·8)	6·2 (6·5)	1·8 (3·3)
Third (>16·0%)	6643	624 (9·4%)	0·34 (0·43)	21·6 (15·3)	11·6 (10·0)	6·0 (6·1)	1·8 (3·0)

Data are n or n (%), where n=number of singleton births, or mean (SD).

* Excluding births with missing information on this variable (table 1).

**Table 3:** Proportion of the study population exposed to smoke waves during pregnancy (N=20 034)

	Smoke wave duration		
	2 days	3 days	≥4 days

Smoke wave intensity, PM_2·5_ concentration			
≥2·5 μg/m^3^	16 140 (80·6%)	11 282 (56·3%)	7874 (39·3%)
≥5·0 μg/m^3^	12 942 (64·6%)	7458 (37·2%)	4108 (20·5%)
≥10·0 μg/m^3^	4864 (24·3%)	2557 (12·8%)	1210 (6·0%)

Data are number of singleton births (%), where the denominator is 20 034.

## Data Availability

Select de-identified data from the ECHO Program are available through the Data and Specimen Hub (DASH) of the Eunice Kennedy Shriver National Institute of Child Health and Human Development (NICHD; https://echochildren.org/dash). Information on study data not available on DASH, such as some Indigenous datasets, can be found on the ECHO Cohort DASH webpage. The data are de-identified and usable for secondary analysis, and researchers must provide details about the study, funding source, principal investigator, and authorised representatives. Additionally, the submission must include the NICHD DASH Data Use Agreement and, if required by the requested study, institutional review board approval for the data request. Analytical code is available at https://github.com/arsherris/wildfire-PTB-ECHO.

## Research in context

### Evidence before this study

We searched PubMed and Google Scholar for epidemiological studies published in English between Jan 1, 1970, and July 1, 2024, using the key words: “wildfire”, “wildland fire”, “preterm birth”, and “birth outcomes”. We identified seven previous epidemiological studies focused on associations between wildfire-specific PM_2·5_ or overall wildfire exposure during pregnancy and preterm birth. The studies used various exposure assessment methods paired with administrative birth records from regions including California, Colorado, other southwestern US states, Brazil, and Australia. All previous studies identified positive associations between average wildfire PM_2·5_ (or days with wildfire PM_2·5_) and preterm birth, although with some variation in the magnitude of associations. Data gaps that we identified included the need for nationwide US studies with diverse populations; more robust assessment of potential confounders and precision variables not available from administrative records; a clearer understanding of the roles of wildfire PM_2·5_ exposure intensity, duration, and timing during pregnancy; and investigation into potential regional and subgroup vulnerabilities.

### Added value of this study

This study investigated the association between wildfire PM_2·5_ and preterm birth within a large, prospective, and geographically diverse US cohort (the National Institutes of Health ECHO Program, from which we assessed 20 034 births during 2006–20 across all 48 contiguous states and the District of Columbia). We used a sophisticated machine learning model for estimates of daily census tract-level wildfire PM_2·5_ exposure, which we linked to longitudinal residential histories. Distinctive aspects of this study include the evaluation of exposure metrics such as smoke days at varying intensity thresholds (wildfire PM_2·5_ ≥2·5, ≥5·0, and ≥10·0 μg/m^3^), and smoke waves (consecutive smoke days meeting these thresholds) to capture the episodic nature of wildfire PM_2·5_ exposure. Nationwide, estimates of association between wildfire PM_2·5_ exposure metrics and preterm birth were in the positive direction but included the null. For the US West region (N=5807), estimates showed significantly increased odds of preterm birth associated with wildfire PM_2·5_ exposures. The findings also suggested an exposure–response relationship, with moderate-to-high-intensity wildfire PM_2·5_ smoke days and longer-duration, moderate-to-high-intensity smoke waves identified as risk factors for preterm birth. We also identified potentially critical windows of exposure in mid-pregnancy (for low-to-moderate-intensity smoke days) and late pregnancy (for high-intensity smoke days). The study incorporated detailed individual-level data and residential histories, allowing for comprehensive covariate adjustment, including for factors such as prepregnancy BMI and substance use during pregnancy.

### Implications of all the available evidence

The findings of this study, combined with existing evidence, suggest that exposure to wildfire PM_2·5_, particularly at high intensities and long durations, is a risk factor for preterm birth. This risk is especially pertinent in the US West, where clear associations were observed and where average wildfire PM_2·5_ concentrations are highest. Given the historical and projected increase in wildfire activity due to climate change, these findings highlight a growing public health concern for pregnant individuals. For practice and policy, this research supports the need for public health interventions aimed at reducing exposure to wildfire smoke during pregnancy, such as targeted advisories, promoting behaviours to reduce wildfire smoke exposure, and community-level mitigation strategies, especially during high-intensity and long-duration wildfire smoke events. Future research should continue to explore regional differences in susceptibility, the effect of co-exposures such as extreme heat, the specific components of wildfire smoke driving toxicity, and the effectiveness of intervention strategies. Further investigation into the critical windows of vulnerability based on exposure intensity, and potential differential susceptibility (eg, by infant sex), is also warranted.
