# Supplementary material for: Wildfire-specific fine particulate matter and preterm birth: a US ECHO Cohort analysis
Source: Lancet Planet Health. Author manuscript; Available in PMC 2026 Feb 9. (PMC12885132; doi:10.1016/j.lanplh.2025.101324)
Supplement: 1 [file NIHMS2133758-supplement-1.pdf]

THE LANCET
Planetary Health

## Supplementary appendix

This appendix formed part of the original submission and has been peer reviewed.
We post it as supplied by the authors.

Supplement to: Sherris AR, Dearborn LC, Goin DE, et al. Wildfire-specific fine particulate matter and preterm birth: a US ECHO Cohort analysis. *Lancet Planet Health* 2025. https://doi.org/10.1016/j.lanplh.2025.101324

# Supplementary Appendix

## Wildfire-specific fine particulate matter and preterm birth: a US ECHO Cohort analysis

### Authors


1. Allison R. Sherris, Department of Environmental and Occupational Health Sciences, University of Washington, Seattle, WA, USA
2. Logan C. Dearborn, Department of Environmental and Occupational Health Sciences, University of Washington, Seattle, WA, USA
3. Dana E. Goin, Mailman School of Public Health, Columbia University, New York, NY, USA
4. Christine T. Loftus, Department of Environmental and Occupational Health Sciences, University of Washington, Seattle, WA, USA
5. Adam A. Szpiro, Department of Biostatistics, University of Washington, Seattle, WA, USA
6. Joan A. Casey, Department of Environmental and Occupational Health Sciences, University of Washington, Seattle, WA, USA
7. Sindana D. Ilango, Department of Epidemiology, University of Washington, Seattle, WA, USA
8. Jyoti Angal, Department of Pediatrics, Avera Research Institute University of South Dakota School of Medicine, Sioux Falls, SD, USA
9. Deborah H. Bennett, Department of Public Health Sciences, University of California, Davis, Davis, CA, USA
10. Miatta A. Buxton, Department of Epidemiology, University of Michigan, Ann Arbor, MI, USA
11. Carlos A. Camargo, Jr., Department of Emergency Medicine, Massachusetts General Hospital, Harvard Medical School, Boston, MA, USA
12. Kecia N. Carroll, Pediatrics and Environmental Medicine, The Icahn School of Medicine at Mount Sinai School, NY, USA
13. Marissa L. Childs, Department of Environmental and Occupational Health Sciences, University of Washington, Seattle, WA, USA
14. Camille Cioffi, Prevention Science Institute, University of Oregon, Eugene, OR, USA
15. Lisa A. Croen, Division of Research, Kaiser Permanente Northern California, Kaiser Permanente, Pleasanton, CA, USA
16. Dana Dabelea, Lifecourse Epidemiology of Adiposity and Diabetes (LEAD) Center, University of Colorado Anschutz Medical Campus, University of Colorado School of Medicine, Aurora, CO, USA
17. Stephanie M. Eick, Gangarosa Department of Environmental Health, Rollins School of Public Health, Emory University, Atlanta, GA, USA
18. Shohreh F. Farzan, Department of Population and Public Health Sciences, University of Southern California , Los Angeles, CA, USA
19. Assiamira Ferrara, Division of Research, Kaiser Permanente Northern California, Kaiser Permanente, Pleasanton, CA, USA
20. Erika Garcia, Department of Population and Public Health Sciences, University of Southern California, Los Angeles, CA, USA
21. Alison Gemmill, Department of Population, Family and Reproductive Health, Johns Hopkins University Bloomberg School of Public Health, Baltimore, MD, USA
22. Frank Gilliland, Department of Population and Public Health Sciences, University of Southern California, Los Angeles, CA, USA
23. Rima Habre, Department of Population and Public Health Sciences, University of Southern California, Los Angeles, CA , USA
24. Irva Hertz-Picciotto, Department of Public Health Sciences, University of California, Davis, Davis, CA, USA
25. Alison E. Hipwell, Department of Psychiatry, Psychology, and Clinical & Translational Science, University of Pittsburgh, Pittsburgh, PA, USA
26. Deborah Hirtz, Department of Neurological Sciences and Pediatrics, University of Vermont School of Medicine, Burlington, VT, USA
27. Margaret R. Karagas, Department of Epidemiology, Geisel School of Medicine, Dartmouth College, Hanover, NH, USA
28. Daphne Koinis-Mitchell, Department of Pediatrics, Brown University, Providence, RI, USA
29. Amii M. Kress, Department of Epidemiology, Johns Hopkins University Bloomberg School of Public Health, Baltimore, MD, USA
30. Leslie D Leve, Prevention Science Institute, University of Oregon, Eugene, OR, USA
31. Donghai Liang, Gangarosa Department of Environmental Health, Rollins School of Public Health, Emory University, Atlanta, GA, USA

32. Kristen Lyall, AJ Drexel Autism Institute, Drexel University, Philadelphia, PA, USA
33. Lacey A. McCormack, Avera Research Institute, Avera McKennan Hospital & University Health Center, Sioux Falls, SD, USA
34. Cindy T. McEvoy, Department of Pediatrics, Oregon Health & Science University, Portland, OR, USA
35. Hooman Mirzakhani, Channing Division of Network Medicine, Brigham and Women's Hospital and Harvard Medical School, Harvard Medical School, Boston, MA, USA
36. Rachel Morello-Frosch, Department of Environmental Science, Policy and Management and School of Public Health, University of California, Berkeley, Berkeley, CA , USA
37. Zhongzheng Niu, Department of Population and Public Health Science, University of Southern California, Los Angeles, CA, USA
38. Thomas G. O'Connor, Departments of Psychiatry, Neuroscience, Obstetrics and Gynecology, University of Rochester, University of Rochester, Rochester, NY, USA
39. Alicia K. Peterson, Division of Research , Kaiser Permanente Northern California , Pleasanton, CA, USA
40. Rebecca J. Schmidt, Department of Public Health Sciences; MIND Institute, University of California, Davis; Davis, CA, USA
41. Catherine J. Karr, Departments of Pediatrics and Environmental and Occupational Health Sciences, University of Washington, Seattle, WA, USA
42. Amy M. Padula, Department of Obstetrics, Gynecology & Reproductive Sciences, University of California, San Francisco, San Francisco, CA, USA
43. For the ECHO Cohort Consortium*

*See Table S7 for full list of collaborators

## Contents

Table S1. Methods of determining gestational age in the study population. 3

Table S2. Distribution of daily $PM_{2.5}$ on smoke days in the study population ($\mu g/m^3$) 3

Table S3. Cohort sites included in the study population and locations of recruitment sites 4

Table S4. Characteristics of the restricted study sample 5

Table S5. Associations (OR and 95% CI) between cumulative wildfire $PM_{2.5}$ during pregnancy and PTB, determined by pooled logistic regression 6

Table S6. Associations (coefficient and 95% CI) between entire pregnancy wildfire exposure and gestational age (weeks) with primary model adjustment (Model 1). 7

Table S7. ECHO Cohort Consortium Collaborators 8

Figure S1. Directed Acyclic Graph (DAG), using arrow-on-arrow representation of effect modification (Weinberg 2008). 15

Figure S2. Inclusion flowchart. 16

Figure S3. Correlation between wildfire $PM_{2.5}$ exposure metrics during pregnancy. 17

Figure S4. Correlation between weekly smoke days across pregnancy. 18

Figure S5. Sensitivity analyses of the relationship between wildfire exposure and preterm birth 19

Figure S6. Associations between trimester-specific exposure to mean wildfire $PM_{2.5}$, smoke days, and smoke waves during pregnancy and conditional odds of preterm birth in Trimester 1 (0-13 weeks), Trimester 2 (14-26 weeks), and Trimester 3. 20

**Table S1. Methods of determining gestational age in the study population.**

| Method | Count (%) |
|---|---|
| Neonatal estimate of gestational age at delivery | 8,092 (40.4%) |
| Self-report or report from caregiver | 6,289 (31.4%) |
| Best obstetrical consensus estimate | 2,127 (10.6%) |
| Administratively recorded estimated date of delivery | 1,328 (6.6%) |
| Obstetrical estimate from last menstrual period only | 1,107 (5.5%) |
| Obstetrical estimate from IVF or first or second trimester ultrasound | 1,091 (5.4%) |

**Table S2. Distribution of daily $PM_{2.5}$ on smoke days in the study population (μg/$m^3$).**

| Census region | Mean | SD | P25 | P50 | P75 | P90 | P99 | Max. |
|---|---|---|---|---|---|---|---|---|
| West | 5.6 | 10.3 | 1.7 | 2.9 | 5.2 | 10.5 | 58.4 | 198.3 |
| Midwest | 3.5 | 3.2 | 1.3 | 2.6 | 4.9 | 7.6 | 14.4 | 49.3 |
| South | 4.8 | 4.2 | 1.8 | 3.9 | 6.4 | 10.7 | 17.1 | 90.2 |
| Northeast | 3.7 | 3.9 | 0.8 | 2.3 | 5.4 | 9.2 | 17.1 | 50.7 |
| **Overall** | **4.3** | **6.4** | **1.4** | **2.8** | **5.4** | **9.0** | **26.2** | **198.3** |

**Table S3. Cohort sites included in the study population and locations of recruitment sites**

| Cohort ID | Cohort Name | N | Birth years | Recruitment site locations |
|---|---|---|---|---|
| 10402 | 43rd Multicenter Airway Research Collaboration (MARC-43) | 275 | 2013-2017 | Boston, MA; Philadelphia, PA; Louisville, KY; Phoenix, AZ |
| 10601 | Healthy Start | 841 | 2010-2014 | Aurora, CO |
| 10703 | MINNIE | 402 | 2018-2020 | Denver, CO |
| 10801 | Boricua Youth Study (BYS) | 129 | 2010-2020 | New York, NY |
| 10901 | Atlanta ECHO Cohort of Emory University | 287 | 2014-2020 | Atlanta, GA |
| 11001 | Safe Passage Study (PASS) | 2,169 | 2008-2020 | Rapid City, SD; Sioux Fans, SD |
| 11201 | Pregnancy Environment and Lifestyle study (PETALS) | 1,115 | 2014-2019 | Richmond, CA; Union City, CA; San Leandro, CA; San Jose, CA |
| 11202 | Kaiser Permanente Research Bank (KPRB) | 641 | 2014-2018 | California (Bay Area and Central Valley) |
| 11401 | Maternal and Development Risks from Environmental and social stressors (MADRES) | 383 | 2016-2020 | Los Angeles, CA |
| 11601 | Revisiting CHildhood Autism Risks from Genes and the Environment Study (ReCHARGE) | 337 | 2006-2014 | Sacramento, CA; Los Angeles, CA |
| 11701 | Pittsburgh Gins study (PGS) | 156 | 2012-2020 | Pittsburgh, PA |
| 11801 | New Hampshire Birth Cohort study (NHBCS) | 2,006 | 2009-2020 | Lebanon, NH; Concord, NH; Warner, NH |
| 11901 | Conditions Affecting Neurocognitive Development and Learning in Early Childh00d (CANDLE) | 815 | 2007-2011 | Memphis, TN |
| 11903 | The Global Alliance to Prevent Prematurity and stillbirth (GAPPS) | 503 | 2011-2017 | Seattle, WA; Yakima, WA |
| 12100 | Early Growth and Development Study Pediatric Cohort | 245 | 2006-2015 | Various locations (CA, VA, NJ, TX, OR, WA, FL, UT, IL, MD, PA) |
| 12301 | Vitamin D Antenatal Asthma Reduction TOA (VDAART) | 393 | 2010-2012 | Boston, MA; San Diego, CA, St. Louis, MO |
| 12401 | Vitamin C to Decrease Effects of Smoking in Pregnancy on Infant Lung Function (VCSIP) | 194 | 2013-2016 | Indianapolis, IN; Portland, OR; Vancouver, WA |
| 12402 | In-Utero Smoke, Vitamin C, and Newborn Lung Function | 115 | 2007-2011 | Portland, OR; Vancouver, WA |
| 12514 | Infant Brain Imaging Study (IBIS) | 122 | 2007-2017 | Philadelphia, PA; Chapel Hill, NC; Seattle, WA |
| 12601 | Rochester | 179 | 2016-2020 | Rochester, NY; Pittsburgh, PA |
| 12602 | Magee | 161 | 2017-2020 | Rochester, NY; Pittsburgh, PA |
| 12901 | Archive for Research in Child Health (ARCH) | 463 | 2008-2017 | Lansing, MI |
| 12902 | Michigan Archive for Research in Child Health (MARCH) | 582 | 2018-2020 | Michigan (statewide) |
| 13102 | Chemicals in our Bodies (CIOB) | 454 | 2014-2020 | San Francisco, CA |
| 13200 | Utah Children's Project | 690 | 2006-2018 | Salt Lake City, UT; Logan, UT |
| 13301 | The NYU Children's Health and Environment Study (NYU CHES) | 1,877 | 2016-2020 | New York, NY |
| 13501 | Asthma Coalition on Community, Environment & Social Stress (ACCESS) | 163 | 2006-2008 | Boston, MA; Roxbury, MA; Brockton, MA; Dorchester, MA |
| 13502 | PRogramming of Intergenerational Stress Mechanisms (PRISM) | 864 | 2011-2020 | East Boston, MA; Boston, MA; New York, NY |
| 13503 | First 1000 Days | 2,959 | 2011-2020 | Falls Church, VA |
| 20101 | The Infant Development and the Environment Study (TIDES) | 514 | 2011-2013 | Seattle, WA, San Francisco, CA; Minneapolis, MN; Rochester, NY |

**Table S4. Characteristics of the restricted study sample**

| | Preterm (N=1329) | Term (N=14898) | Overall (N=16227) |
|---|---|---|---|
| **Child sex** | | | |
| Male | 692 (52.1%) | 7604 (51.0%) | 8296 (51.1%) |
| Female | 637 (47.9%) | 7287 (48.9%) | 7924 (48.8%) |
| Missing | 0 (0%) | 7 (0.0%) | 7 (0.0%) |
| **Age of the pregnant individual** | | | |
| Mean (SD) | 31.1 (6.08) | 30.8 (5.45) | 30.9 (5.51) |
| Missing | 1 (0.1%) | 7 (0.0%) | 8 (0.0%) |
| **Race of the pregnant individual** | | | |
| White | 797 (60.0%) | 9770 (65.6%) | 10567 (65.1%) |
| Black | 186 (14.0%) | 1398 (9.4%) | 1584 (9.8%) |
| Asian, Native Hawaiian, or other Pacific Islander | 98 (7.4%) | 1111 (7.5%) | 1209 (7.5%) |
| American Indian or Alaska Native | 51 (3.8%) | 320 (2.1%) | 371 (2.3%) |
| More than one race or Other Race | 105 (7.9%) | 1251 (8.4%) | 1356 (8.4%) |
| Missing | 92 (6.9%) | 1048 (7.0%) | 1140 (7.0%) |
| **Ethnicity of the pregnant individual** | | | |
| Hispanic | 301 (22.6%) | 3236 (21.7%) | 3537 (21.8%) |
| Non-Hispanic | 1007 (75.8%) | 11349 (76.2%) | 12356 (76.1%) |
| Missing | 21 (1.6%) | 313 (2.1%) | 334 (2.1%) |
| **Educational level of the pregnant individual** | | | |
| High school degree or less | 322 (24.2%) | 3061 (20.5%) | 3383 (20.8%) |
| Some college, Associate's degree, or Trade school | 309 (23.3%) | 2724 (18.3%) | 3033 (18.7%) |
| Bachelor's degree | 301 (22.6%) | 3782 (25.4%) | 4083 (25.2%) |
| Postgraduate degree | 243 (18.3%) | 3379 (22.7%) | 3622 (22.3%) |
| Missing | 154 (11.6%) | 1952 (13.1%) | 2106 (13.0%) |
| **Parity** | | | |
| 1 | 507 (38.1%) | 5694 (38.2%) | 6201 (38.2%) |
| 2 | 399 (30.0%) | 5024 (33.7%) | 5423 (33.4%) |
| 3 or more | 347 (26.1%) | 3254 (21.8%) | 3601 (22.2%) |
| Missing | 76 (5.7%) | 926 (6.2%) | 1002 (6.2%) |
| **Pregnancy tobacco use** | | | |
| Yes | 123 (9.3%) | 1040 (7.0%) | 1163 (7.2%) |
| No | 1117 (84.0%) | 12998 (87.2%) | 14115 (87.0%) |
| Missing | 89 (6.7%) | 860 (5.8%) | 949 (5.8%) |
| **Pregnancy alcohol consumption** | | | |
| Yes | 210 (15.8%) | 2608 (17.5%) | 2818 (17.4%) |
| No | 927 (69.8%) | 10483 (70.4%) | 11410 (70.3%) |
| Missing | 192 (14.4%) | 1807 (12.1%) | 1999 (12.3%) |
| **Census region** | | | |
| Midwest | 260 (19.6%) | 2578 (17.3%) | 2838 (17.5%) |
| Northeast | 283 (21.3%) | 3721 (25.0%) | 4004 (24.7%) |
| South | 340 (25.6%) | 3819 (25.6%) | 4159 (25.6%) |
| West | 446 (33.6%) | 4780 (32.1%) | 5226 (32.2%) |
| **Conception season** | | | |
| Autumn | 342 (25.7%) | 3969 (26.6%) | 4311 (26.6%) |
| Spring | 342 (25.7%) | 3514 (23.6%) | 3856 (23.8%) |
| Summer | 319 (24.0%) | 3891 (26.1%) | 4210 (25.9%) |
| Winter | 326 (24.5%) | 3524 (23.7%) | 3850 (23.7%) |
| **Birth year** | | | |
| 2006-2009 | 116 (8.7%) | 1092 (7.3%) | 1208 (7.4%) |
| 2010-2013 | 403 (30.3%) | 4179 (28.1%) | 4582 (28.2%) |
| 2014-2017 | 506 (38.1%) | 5735 (38.5%) | 6241 (38.5%) |
| 2018-2021 | 304 (22.9%) | 3892 (26.1%) | 4196 (25.9%) |

**Table S5. Associations (OR and 95% CI) between cumulative mean wildfire $PM_{2.5}$ (per 1 μg/m$^3$ increase) and cumulative smoke days and smoke waves (per additional smoke day or smoke wave) during pregnancy and PTB, determined by pooled logistic regression. Models included age, race, and ethnicity of the pregnant individual; child sex; neighborhood poverty rate; season of conception, year of birth splines (4 df), spatial splines (10 df), and cohort random intercept.**

| | Nationwide ECHO sites | | | Western U.S. ECHO sites | | |
|---|---|---|---|---|---|---|
| | Full sample | Restricted sample | | Full sample | Restricted sample | |
| Exposure | Model 1 (N = 20,034) | Model 1 (N = 16,227) | Model 2 (N = 16,227) | Model 1 (N = 5,807) | Model 1 (N = 5,226) | Model 2 (N = 5,226) |
| **Mean wildfire $PM_{2.5}$** | 1.069 (0.964, 1.187) | 1.045 (0.938, 1.165) | 1.080 (0.968, 1.204) | 1.139 (1.001, 1.296) | 1.140 (0.999, 1.301) | 1.178 (1.029, 1.349) |
| **Smoke days** | | | | | | |
| Any | 1.002 (0.998, 1.006) | 1.001 (0.997, 1.006) | 1.002 (0.997, 1.007) | 1.005 (0.997, 1.013) | 1.003 (0.995, 1.011) | 1.006 (0.998, 1.014) |
| ≥ 2.5 μg/m$^3$ | 1.003 (0.998, 1.009) | 1.002 (0.995, 1.008) | 1.003 (0.997, 1.010) | 1.009 (0.999, 1.019) | 1.008 (0.998, 1.018) | 1.010 (1.000, 1.021) |
| ≥ 5.0 μg/m$^3$ | 1.007 (0.998, 1.016) | 1.004 (0.995, 1.014) | 1.007 (0.997, 1.016) | 1.018 (1.003, 1.032) | 1.016 (1.002, 1.031) | 1.020 (1.005, 1.035) |
| ≥ 10 μg/m$^3$ | 1.010 (0.993, 1.028) | 1.007 (0.989, 1.026) | 1.011 (0.993, 1.030) | 1.030 (1.006, 1.054) | 1.031 (1.007, 1.056) | 1.035 (1.011, 1.061) |
| **Smoke waves** | | | | | | |
| ≥ 2.5 μg/m$^3$ | | | | | | |
| 2 days | 1.004 (0.978, 1.031) | 0.993 (0.964, 1.023) | 1.001 (0.973, 1.031) | 1.013 (0.964, 1.064) | 1.005 (0.955, 1.057) | 1.018 (0.968, 1.071) |
| 3 days | 0.997 (0.957, 1.039) | 1.001 (0.956, 1.047) | 1.008 (0.964, 1.054) | 1.036 (0.967, 1.110) | 1.021 (0.949, 1.098) | 1.038 (0.966, 1.116) |
| 4+ days | 1.010 (0.953, 1.071) | 0.992 (0.932, 1.057) | 1.012 (0.951, 1.078) | 1.077 (0.977, 1.187) | 1.064 (0.961, 1.178) | 1.089 (0.983, 1.206) |
| ≥ 5.0 μg/m$^3$ | | | | | | |
| 2 days | 1.016 (0.979, 1.055) | 1.005 (0.964, 1.049) | 1.013 (0.972, 1.056) | 1.075 (1.000, 1.154) | 1.062 (0.987, 1.144) | 1.077 (0.999, 1.161) |
| 3 days | 1.038 (0.978, 1.102) | 1.042 (0.979, 1.111) | 1.052 (0.988, 1.121) | 1.106 (0.992, 1.233) | 1.108 (0.991, 1.239) | 1.134 (1.013, 1.271) |
| 4+ days | 1.033 (0.946, 1.129) | 1.015 (0.924, 1.114) | 1.036 (0.945, 1.137) | 1.185 (1.044, 1.347) | 1.176 (1.031, 1.341) | 1.200 (1.050, 1.373) |
| ≥ 10 μg/m$^3$ | | | | | | |
| 2 days | 1.019 (0.948, 1.096) | 1.017 (0.937, 1.103) | 1.033 (0.953, 1.119) | 1.107 (0.978, 1.253) | 1.116 (0.984, 1.266) | 1.129 (0.992, 1.285) |
| 3 days | 1.019 (0.901, 1.152) | 1.007 (0.883, 1.149) | 1.034 (0.908, 1.177) | 1.161 (0.986, 1.367) | 1.165 (0.986, 1.377) | 1.198 (1.010, 1.421) |
| 4+ days | 1.107 (0.942, 1.300) | 1.083 (0.916, 1.282) | 1.119 (0.948, 1.321) | 1.232 (1.029, 1.475) | 1.245 (1.037, 1.495) | 1.285 (1.065, 1.552) |

Note: Model 1 (“primary model”) was adjusted for age, race, and ethnicity of the pregnant individual; child sex; neighborhood poverty rate; season of conception, year of birth splines (4 df), spatial splines (10 df), and cohort random intercept. Model 2 (“extended model”) additionally included parity, pre-pregnancy BMI, self-reported pregnancy tobacco use, self-reported pregnancy alcohol consumption, method of determining gestational age, and education level of the pregnant individual.

**Table S6. Associations (coefficient and 95% CI) between entire pregnancy wildfire exposure and gestational age (weeks) with primary model adjustment (Model 1). Estimates represent a change in gestational age (in weeks) associated with a 1 μg/m$^3$ increase in wildfire $PM_{2.5}$ exposure or an additional smoke day or smoke wave from week 0-32 of gestation.**

| Exposure | Entire pregnancy (0-32 weeks) |
|---|---|
| **Mean smoke $PM_{2.5}$** | 0.001 (-0.048, 0.050) |
| **Smoke days** | |
| Any | -0.002 (-0.004, 0.000) |
| ≥ 2.5 μg/m$^3$ | -0.002 (-0.005, 0.001) |
| ≥ 5.0 μg/m$^3$ | -0.002 (-0.007, 0.002) |
| ≥ 10 μg/m$^3$ | -0.001 (-0.009, 0.008) |
| **Smoke waves** | |
| ≥ 2.5 μg/m$^3$ | |
| 2 days | -0.007 (-0.019, 0.006) |
| 3 days | -0.006 (-0.025, 0.013) |
| 4+ days | -0.008 (-0.035, 0.019) |
| ≥ 5.0 μg/m$^3$ | |
| 2 days | -0.002 (-0.020, 0.016) |
| 3 days | -0.007 (-0.036, 0.021) |
| 4+ days | -0.020 (-0.062, 0.021) |
| ≥ 10 μg/m$^3$ | |
| 2 days | -0.004 (-0.039, 0.030) |
| 3 days | -0.008 (-0.066, 0.050) |
| 4+ days | -0.057 (-0.137, 0.022) |

Note: Models were adjusted for age, race, and ethnicity of the pregnant individual; child sex; neighborhood poverty rate; season of conception, year of birth splines (4 df), spatial splines (10 df), and cohort random intercept.

**Table S7. ECHO Cohort Consortium Collaborators**

| Name | Institution | ECHO Cohort Study Site or Core Name and Grant Number |
|---|---|---|
| Brian P. Smith | Duke Clinical Research Institute, Duke University School of Medicine | U2COD023375 (Coordinating Center) |
| Kristin L. Newby | | |
| Linda Adair | Gillings School of Global Public Health, University of North Carolina at Chapel Hill | U2COD023375 (Coordinating Center) |
| Lisa P. Jacobson | Johns Hopkins University, Bloomberg School of Public Health | U24OD023382 (Data Analysis Center) |
| Diane Catellier | Research Triangle Institute | |
| Monica McGrath | Johns Hopkins University, Bloomberg School of Public Health | |
| Christian Douglas | Research Triangle Institute | |
| Priya Duggal | Johns Hopkins University, Bloomberg School of Public Health | |
| Emily Knapp | | |
| Amii Kress | | |
| Courtney K. Blackwell | Feinberg School of Medicine, Northwestern University | U24OD023319 with co-funding from the Office of Behavioral and Social Science Research (Measurement Core) |
| Maxwell A. Mansolf | | |
| Jin-Shei Lai | | |
| Emily Ho | | |
| David Cella | | |
| Richard Gershon | | |
| Michelle L. Macy | Feinberg School of Medicine, Northwestern University and Ann & Robert H. Lurie Children's Hospital of Chicago | |
| Suman R. Das | Vanderbilt University Medical Center | U24OD035523 (Lab Core) |
| Jane E. Freedman | | |
| Simon A. Mallal | | |
| John A. McLean | | |
| Ravi V. Shah | | |
| Meghan H. Shilts | | |
| Akram N. Alshawabkeh | Northeastern University | UG3/UH3OD023251 (Akram Alshawabkeh) |
| Jose F. Cordero | University of Georgia | |
| John Meeker | University of Michigan | |
| Leonardo Trasande | NYU Grossman School of Medicine | UG3/UH3OD023305 (Leonardo Trasande) |
| Carlos A. Camargo | Massachusetts General Hospital, Harvard Medical School | UG3/UH3OD023253 (Carlos Camargo) |
| Kohei Hasegawa | | |
| Zhaozhong Zhu | | |
| Ashley F. Sullivan | | |
| Dana Dabelea | University of Colorado Anschutz Medical Campus | UG3/UH3OD023248 and UG3OD035526 (Dana Dabelea) |
| Wei Perng | | UG3/UH3OD023248 (Dana Dabelea) |
| Traci A. Bekelman | | |
| Greta Wilkening | | |
| Sheryl Magzamen | Colorado School of Public Health, Colorado State University | UG3OD035526 (Dana Dabelea) |
| Brianna F. Moore | University of Colorado Anschutz Medical Campus | |
| Anne P. Starling | University of North Carolina at Chapel Hill | |
| Deborah J. Rinehart | Denver Health and Hospital Authority | |
| Daphne Koinis Mitchell | Rhode Island Hospital, The Alpert Medical School of Brown University | UG3/UH3OD023313 (Daphne Koinis Mitchell) |
| Viren D'Sa | | |
| Sean C.L. Deoni | Bill & Melinda Gates Foundation | |

| | | |
|---|---|---|
| Hans-Georg Mueller | University of California, Davis | |
| Cristiane S. Duarte | Columbia University - NYSPI | UH3OD023328 (Cristiane Duarte) |
| Catherine Monk | | |
| Glorisa Canino | University of Puerto Rico, School of Medicine | |
| Jonathan Posner | Duke University School of Medicine, Duke Psychiatry & Behavioral Sciences | |
| Tenneill Murray | Columbia University - NYSPI | |
| Claudia Lugo-Candelas | | |
| Anne L. Dunlop | Emory University School of Medicine | UH3OD023318 (Anne Dunlop) |
| Patricia A. Brennan | Emory University | |
| Christine Hockett | Avera Research Institute; University of South Dakota School of Medicine | UG3/UH3OD023279 (Amy Elliott) |
| Amy Elliott | | |
| Assiamira Ferrara | Kaiser Permanente Northern California | UG3/UH3OD023289 (Assiamira Ferrara) |
| Lisa A. Croen | | UG3/UH3OD023342 (Kristen Lyall), UG3/UH3OD023290 (Julie Herbstman) |
| Monique M. Hedderson | | UG3/UH3OD023289 (Assiamira Ferrara), UG3OD035540 (Monique Marie Hedderson) |
| John Ainsworth | University of Manchester | UG3/UH3OD023282 (James Gern) |
| Leonard B. Bacharier | Vanderbilt University Medical Center | |
| Casper G. Bendixsen | Marshfield Clinic Research Institute | |
| James E. Gern | University of Wisconsin School of Medicine and Public Health | UG3/UH3OD023282 (James Gern), UG3OD035509 (Anne Marie Singh) |
| Diane R. Gold | Brigham and Women's Hospital; Harvard Medical School | UG3/UH3OD023282 (James Gern) |
| Tina V. Hartert | Vanderbilt University Medical Center | UG3/UH3OD023282 (James Gern), UG3OD035516 and UG3OD035517 (Tina Hartert) |
| Daniel J. Jackson | University of Wisconsin School of Medicine and Public Health | UG3/UH3OD023282 (James Gern) |
| Christine C. Johnson | Henry Ford Health | UG3/UH3OD023282 (James Gern), UG3OD035518 (Jennifer Straughen) |
| Christine L.M. Joseph | | UG3/UH3OD023282 (James Gern) |
| Meyer Kattan | Columbia University Medical Center | |
| Gurjit K. Khurana Hershey | Cincinnati Children's Hospital Medical Center | |
| Robert F. Lemanske, Jr. | University of Wisconsin School of Medicine and Public Health | |
| Susan V. Lynch | University of California | |
| Rachel L. Miller | Icahn School of Medicine at Mount Sinai | UG3/UH3OD023282 (James Gern), UG3/UH3OD023290 (Julie Herbstman) |
| George T. O'Connor | Boston University School of Medicine | UG3/UH3OD023282 (James Gern) |
| Carole Ober | University of Chicago | UG3/UH3OD023282 (James Gern), UG3OD035509 (Anne Marie Singh) |
| Dennis Ownby | Henry Ford Health | UG3/UH3OD023282 (James Gern) |
| Katherine Rivera-Spoljaric | Washington University School of Medicine | UG3/UH3OD023282 (James Gern), UG3OD035521 (Katherine Rivera-Spoljaric) |
| Patrick H. Ryan | University of Cincinnati | UG3/UH3OD023282 (James Gern), UG3OD035509 (Anne Marie Singh) |
| Christine M. Seroogy | University of Wisconsin School of Medicine and Public Health | UG3/UH3OD023282 (James Gern) |
| Anne Marie Singh | University of Wisconsin School of Medicine and Public Health | UG3/UH3OD023282 (James Gern), UG3OD035509 (Anne Marie Singh) |
| Robert A. Wood | Johns Hopkins University School of Medicine | UG3/UH3OD023282 (James Gern) |
| Edward M. Zoratti | Henry Ford Health | UG3/UH3OD023282 (James Gern), UG3OD035518 (Jennifer Straughen) |
| Rima Habre | University of Southern California | UH3OD023287 (Carrie Breton) |
| Shohreh Farzan | | |
| Frank D. Gilliland | | |
| Irva Hertz-Picciotto | University of California, Davis | UG3/UH3OD023365 (Irva Hertz-Picciotto), UG3OD035550 (Rebecca Schmidt) |
| Deborah H. Bennett | | |

| | | |
|---|---|---|
| Julie B. Schweitzer | | UG3/UH3OD023365 (Irva Hertz-Picciotto) |
| Rebecca J. Schmidt | | UG3/UH3OD023365 (Irva Hertz-Picciotto), UG3/UH3OD023342 (Kristen Lyall), UG3OD035550 (Rebecca Schmidt) |
| Janine M. LaSalle | | UG3/UH3OD023365 (Irva Hertz-Picciotto), UG3OD035550 (Rebecca Schmidt) |
| Alison E. Hipwell | University of Pittsburgh | UG3/UH3OD023244 (Alison Hipwell) |
| Kate E. Keenan | University of Chicago | |
| Catherine J. Karr | University of Washington | UH3OD023271 and UG3OD035528 (Catherine Karr) |
| Nicole R. Bush | University of California, San Francisco | UH3OD023271 (Catherine Karr), UG3OD035519 (Qi Zhao) |
| Kaja Z. LeWinn | | |
| Sheela Sathyanarayana | University of Washington and Seattle Children's Research Institute | UH3OD023271 (Catherine Karr), UG3OD035508 (Sheela Sathyanarayana) |
| Qi Zhao | University of Tennessee Health Science Center | UH3OD023271 (Catherine Karr), UG3OD035519 (Qi Zhao) |
| Frances Tylavsky | University of Tennessee Health Science Center | UH3OD023271 (Catherine Karr) |
| Kecia N. Carroll | Icahn School of Medicine at Mount Sinai | UH3OD023271 (Catherine Karr), UG3/UH3OD023337 (Rosalind Wright) |
| Christine T. Loftus | University of Washington | UH3OD023271 (Catherine Karr) |
| Leslie D. Leve | University of Oregon | UG3/UH3OD023389 (Leslie Leve) |
| Jody M. Ganiban | George Washington University | |
| Jenae M. Neiderhiser | Penn State University | |
| Scott T. Weiss | Brigham and Women's Hospital and Harvard Medical School | UH3OD023268 (Scott Weiss) |
| Augusto A. Litonjua | Golisano Children's Hospital, University of Rochester | |
| Cindy T. McEvoy | Oregon Health & Science University | UG3/UH3OD023288 (Cynthia McEvoy) |
| Eliot R. Spindel | Oregon National Primate Research Center | |
| Robert S. Tepper | Indiana School of Medicine | |
| Craig J. Newschaffer | Penn State | UG3/UH3OD023342 (Kristen Lyall) |
| Kristen Lyall | Drexel University | |
| Heather E. Volk | Johns Hopkins University | |
| Rebecca Landa | Center for Autism and Related Disorders, Kennedy Krieger Institute, Johns Hopkins University | |
| Sally Ozonoff | University of California Davis | |
| Joseph Piven | University of North Carolina | |
| Heather Hazlett | University of North Carolina | |
| Juhi Pandey | Children's Hospital of Philadelphia | |
| Robert Schultz | Children's Hospital of Philadelphia | |
| Steven Dager | University of Washington | |
| Kelly Botteron | Washington University | |
| Daniel Messinger | University of Miami | |
| Wendy Stone | University of Washington | |
| Jennifer Ames | Kaiser Permanente | |
| Thomas G. O'Connor | University of Rochester | UG3/UH3OD023349 (Thomas O'Connor) |
| Richard K. Miller | | |
| Emily Oken | Harvard Pilgrim Health Care Institute and Harvard Medical School | UH3OD023286 and UG3OD035533 (Emily Oken) |
| Michele R. Hacker | Beth Israel Deaconess Medical Center | UG3OD035533 (Emily Oken) |
| Tamarra James-Todd | Harvard Chan School of Public Health | UG3OD035533 (Emily Oken) |
| Michael O'Shea | University of North Carolina School of Medicine | UG3/UH3OD023348 (Mike O'Shea), UH3OD023347 (Barry Lester) |
| Rebecca C. Fry | University of North Carolina Gillings School of Global Public Health | UG3/UH3OD023348 (Mike O'Shea) |

| | | |
|---|---|---|
| Jean A. Frazier | UMASS Chan Medical School | |
| Rachana Singh | Tufts University School of Medicine | |
| Caitlin Rollins | Harvard Medical School | |
| Angela Montgomery | Yale School of Medicine | |
| Ruben Vaidya | University of Massachusetts Chan Medical School-Baystate | |
| Robert M. Joseph | Boston University Chobanian & Avedisian School of Medicine | |
| Lisa K. Washburn | Wake Forest School of Medicine | |
| Semsa Gogcu | Wake Forest School of Medicine; Wake Forest University School of Medicine/Atrium Health Wake Forest | UG3/UH3OD023348 (Mike O'Shea), UG3OD035513 (Annemarie Stroustrup), UH3OD023320 (Judy Aschner) |
| Kelly Bear | ECU Health | UG3/UH3OD023348 (Mike O'Shea) |
| Julie V. Rollins | University of North Carolina School of Medicine | |
| Stephen R. Hooper | | |
| Genevieve Taylor | | |
| Wesley Jackson | | |
| Amanda Thompson | University of North Carolina at Chapel Hill; Gillings School of Global Public Health, University of North Carolina at Chapel Hill | |
| Julie Daniels | | |
| Michelle Hernandez | School of Medicine, University of North Carolina at Chapel Hill | |
| Kun Lu | Gillings School of Global Public Health, University of North Carolina at Chapel Hill | |
| Michael Msall | University of Chicago Medicine: Comer Children's Hospital | |
| Madeleine Lenski | Michigan State University | |
| Rawad Obeid | Beaumont Hospital | |
| Steven L. Pastyrnak | Corewell Health, Helen DeVos Children's Hospital | UG3/UH3OD023348 (Mike O'Shea), UH3OD023347 (Barry Lester) |
| Elizabeth Jensen | Wake Forest University School of Medicine | UG3/UH3OD023348 (Mike O'Shea) |
| Christina Sakai | Mass General Hospital for Children | UG3/UH3OD023348 (Mike O'Shea) |
| Hudson Santos | University of Miami | UG3/UH3OD023348 (Mike O'Shea), UG3OD035542 (Hudson Santos) |
| Jean M. Kerver | Michigan State University, College of Human Medicine | UG3/UH3OD023285 (Jean Kerver) |
| Nigel Paneth | | UG3/UH3OD023285 (Jean Kerver) |
| Charles J. Barone | Henry Ford Health | UG3/UH3OD023285 (Jean Kerver), UG3/UH3OD023282 (James Gern) |
| Michael R. Elliott | University of Michigan | UG3/UH3OD023285 (Jean Kerver) |
| Douglas M. Ruden | Wayne State University | UG3/UH3OD023285 (Jean Kerver) |
| Chris Fussman | Michigan Department of Health and Human Services (MDHHS) | UG3/UH3OD023285 (Jean Kerver) |
| Julie B. Herbstman | Columbia University Mailman School of Public Health | UG3/UH3OD023290 (Julie Herbstman) |
| Amy Margolis | Columbia University Irving Medical Center | UG3/UH3OD023290 (Julie Herbstman) |
| Susan L. Schantz | University of Illinois Urbana-Champaign | UG3/UH3OD023272 (Susan Schantz) |
| Sarah Dee Geiger | | |
| Andrea Aguiar | | |
| Karen Tabb | | |
| Rita Strakovsky | Michigan State University | |
| Tracey Woodruff | University of California, San Francisco | |
| Rachel Morello-Frosch | University of California, Berkeley | |
| Amy Padula | University of California, San Francisco | |
| Joseph B. Stanford | Spencer Fox Eccles School of Medicine, University of Utah | UG3/UH3OD023249 (Joseph Stanford) |
| Christina A. Porucznik | | |
| Angelo P. Giardino | | |
| Rosalind J. Wright | Icahn School of Medicine at Mount Sinai | UG3/UH3OD023337 (Rosalind Wright) |

| | | |
|---|---|---|
| Robert O. Wright | | |
| Brent Collett | University of Washington, Seattle Children's Research Institute | UG3OD035508 (Sheela Sathyanarayana) |
| Nicole Baumann-Blackmore | University of Wisconsin School of Medicine and Public Health | UG3OD035509 (Anne Marie Singh) |
| Ronald Gangnon | University of Wisconsin | |
| Daniel J. Jackson | University of Wisconsin School of Medicine and Public Health | |
| Chris G. McKennan | University of Pittsburgh | |
| Jo Wilson | University of Wisconsin School of Medicine and Public Health | |
| Matt Altman | University of Washington | |
| Judy L. Aschner | Albert Einstein College of Medicine; Hackensack Meridian School of Medicine; Center for Discovery and Innovation | UH3OD023320 and UG3OD035546 (Judy Aschner), UG3OD035513 (Annemarie Stroustrup) |
| Annemarie Stroustrup | Northwell Health, Cohen Children's Medical Center, and the Zucker School of Medicine at Hofstra / Northwell | UH3OD023320 (Judy Aschner), UG3OD035513 (Annemarie Stroustrup) |
| Stephanie L. Merhar | Cincinnati Children's | |
| Paul E. Moore | Vanderbilt University Medical Center | |
| Gloria S. Pryhuber | University of Rochester Medical Center | UH3OD023320 (Judy Aschner) |
| Mark Hudak | University of Florida College of Medicine | |
| Ann Marie Reynolds Lyndaker | University of Buffalo Jacobs School of Medicine and Biomedical Sciences | |
| Andrea L. Lampland | Children's Minnesota | |
| Burton Rochelson | Northwell Health and the Zucker School of Medicine at Hofstra / Northwell | UG3OD035532 (Annemarie Stroustrup) |
| Sophia Jan | Northwell Health, Cohen Children's Medical Center, and the Zucker School of Medicine at Hofstra / Northwell | |
| Matthew J. Blitz | Northwell Health and the Zucker School of Medicine at Hofstra / Northwell | |
| Michelle W. Katzow | | |
| Zenobia Brown | Northwell Health and the Zucker School of Medicine at Hofstra / Northwell | |
| Codruta Chiuzan | Northwell Health, Feinstein Institutes for Medical Research | |
| Timothy Rafael | Northwell Health and the Zucker School of Medicine at Hofstra / Northwell | |
| Dawnette Lewis | | |
| Natalie Meirowitz | | |
| Brenda Poindexter | Children's Healthcare of Atlanta Emory University | UH3OD023320 (Judy Aschner) |
| Tebeb Gebretsadik | Vanderbilt University Medical Center | UG3OD035516 and UG3OD035517 (Tina Hartert) |
| Sarah Osmundson | Vanderbilt University Medical Center | UG3OD035517 (Tina Hartert) |
| Jennifer K. Straughen | Henry Ford Health | UG3OD035518 (Jennifer Straughen) |
| Amy Eapen | | |
| Andrea Cassidy-Bushrow | | UG3/UH3OD023282 (James Gern) |
| Ganesa Wegienka | | |
| Alex Sitarik | | |
| Kim Woodcroft | | UG3OD035518 (Jennifer Straughen), UG3/UH3OD023282 (James Gern) |
| Audrey Urquhart | | |
| Albert Levin | | UG3OD035518 (Jennifer Straughen) |
| Tisa Johnson-Hooper | | |
| Brent Davidson | | UG3/UH3OD023282 (James Gern) |
| Tengfei Ma | | UG3OD035518 (Jennifer Straughen) |
| Emily S. Barrett | Environmental and Occupational Health Sciences Institute, Rutgers University | UG3OD035527 (Emily S Barrett) |
| Martin J. Blaser | Rutgers University | |
| Maria Gloria Dominguez-Bello | | |
| Daniel B. Horton | Robert Wood Johnson Medical School, Rutgers University | |

| | | |
|---|---|---|
| Manuel Jimenez | | |
| Todd Rosen | | |
| Kristy Palomares | Saint Peter's University Hospital | |
| Lyndsay A. Avalos | Kaiser Permanente Northern California | UG3OD035540 (Monique Marie Hedderson) |
| Yeyi Zhu | | |
| Kelly J . Hunt | Medical University of South Carolina | UG3OD035543 (Kelly J Hunt) |
| Roger B. Newman | Medical University of South Carolina | |
| Michael S. Bloom | George Mason University | |
| Mallory H. Alkis | Medical University of South Carolina | |
| James R. Roberts | Medical University of South Carolina | |
| Sunni L. Mumford | University of Pennsylvania Perelman School of Medicine | UG3OD035537 (Sunni L Mumford) |
| Heather H. Burris | Children's Hospital of Philadelphia; University of Pennsylvania Perelman School of Medicine | |
| Sara B. DeMauro | | |
| Lynn M. Yee | Feinberg School of Medicine, Northwestern University | UG3OD035546 (Judy Aschner) |
| Aaron Hamvas | Ann & Robert H. Lurie Children's Hospital, Feinberg School of Medicine, Northwestern University | |
| Antonia F. Olidipo | Hackensack University Medical Center, Hackensack Meridian School of Medicine | |
| Andrew S. Haddad | | |
| Lisa R. Eiland | | |
| Nicole T. Spillane | | |
| Kirin N. Suri | | |
| Stephanie A. Fisher | Feinberg School of Medicine, Northwestern University | |
| Jeffrey A. Goldstein | Feinberg School of Medicine, Northwestern University | |
| Leena B. Mithal | Ann & Robert H. Lurie Children's Hospital, Feinberg School of Medicine, Northwestern University | |
| Raye-Ann O. DeRegnier | Ann & Robert H. Lurie Children's Hospital, Feinberg School of Medicine, Northwestern University | |
| Nathalie L. Maitre | Emory University School of Medicine and Cerebral Palsy Foundation | |
| Ruby H.N. Nguyen | School of Public Health, University of Minnesota | UG3OD035529 (Hong-Ngoc Nguyen) |
| Meghan M. JaKa | HealthPartners Institute | |
| Abbey C. Sidebottom | Allina Health | |
| Michael J. Paidas | University of Miami Miller School of Medicine | UG3OD035542 (Hudson Santos) |
| JoNell E. Potter | | |
| Natale Ruby | | |
| Lunthita Duthely | | |
| Arumugam Jayakumar | | |
| Karen Young | | |
| Isabel Maldonado | University of Miami | |
| Meghan Miller | University of California Davis | UG3OD035550 (Rebecca Schmidt) |
| Jonathan L. Slaughter | Nationwide Children's Hospital and The Ohio State University | UG3OD035536 (Jonathan Slaughter) |
| Sarah A. Keim | | |
| Courtney D. Lynch | The Ohio State University | |
| Kartik K. Venkatesh | | |
| Kristina W. Whitworth | Baylor College of Medicine | UG3OD035544 (Kristina Whitworth) |
| Elaine Symanski | | |
| Thomas F. Northrup | University of Texas Health Science Center at Houston (UTHealth Houston) McGovern Medical School | |
| Hector Mendez-Figueroa | | |
| Ricardo A. Mosquera | | |

| | | |
|---|---|---|
| Margaret R. Karagas | Geisel School of Medicine at Dartmouth | UG3/UH3OD023275 (Margaret Karagas) |
| Juliette C. Madan | Geisel School of Medicine at Dartmouth, Dartmouth Hitchcock Medical Center | |
| Debra M. MacKenzie | College of Pharmacy, University of New Mexico Health Sciences Center | UG3/UH3OD023344 (Debra MacKenzie) |
| Johnnye L. Lewis | | |
| Brandon J. Rennie | University of New Mexico | |
| Bennett L. Leventhal | College of Pharmacy, University of New Mexico Health Sciences Center; University of Chicago | |
| Young Shin Kim | University of California, San Francisco | |
| Somer Bishop | University of California, San Francisco | |
| Sara S. Nozadi | College of Pharmacy, University of New Mexico Health Sciences Center | |
| Li Luo | Comprehensive Cancer Center, University of New Mexico Health Sciences Center | |
| Barry M. Lester | Warren Alpert Medical School of Brown University | UH3OD023347 (Barry Lester) |
| Carmen J. Marsit | Rollins School of Public Health, Emory University | |
| Todd Everson | | |
| Cynthia M. Loncar | Warren Alpert Medical School of Brown University | |
| Elisabeth C. McGowan | | |
| Stephen J. Sheinkopf | Thompson Center for Autism & Neurodevelopment, University of Missouri | |
| Brian S. Carter | Children's Mercy-Kansas City | |
| Jennifer Check | Wake Forest School of Medicine | |
| Jennifer B. Helderman | | |
| Charles R. Neal | University of Hawaii John A Burns School of Medicine | |
| Lynne M. Smith | UCLA Clinical and Translational Science Institute at The Lundquist Institute, Harbor-UCLA Medical Center | |

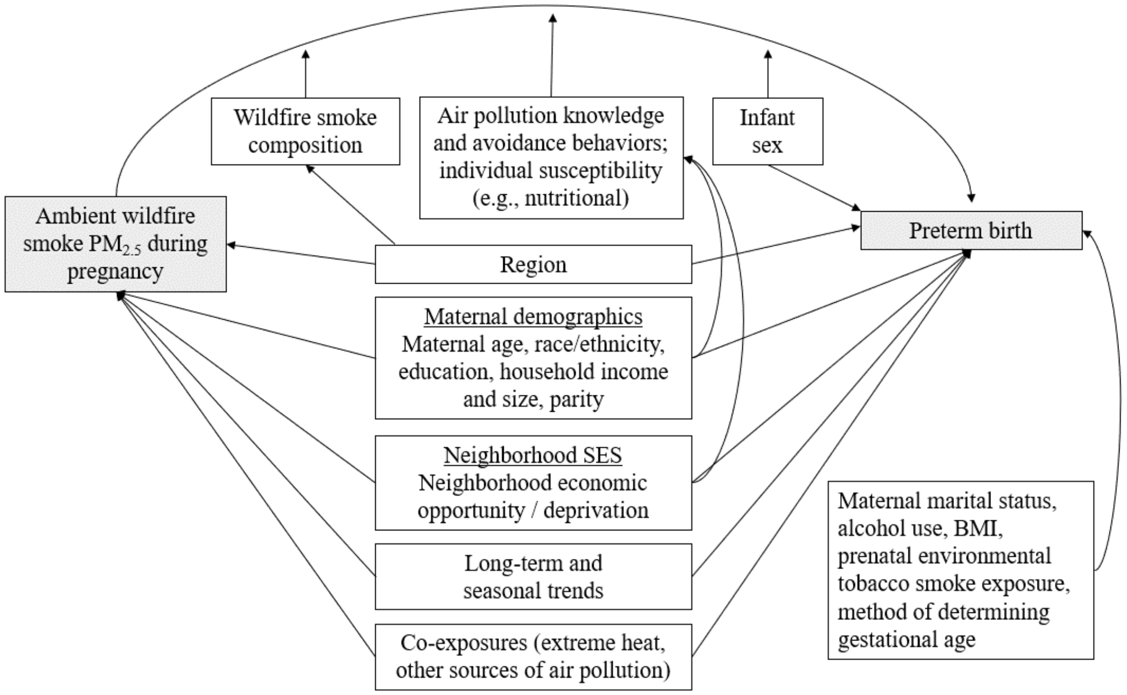


**Figure S1. Directed Acyclic Graph (DAG), using arrow-on-arrow representation of effect modification (Weinberg 2008).**

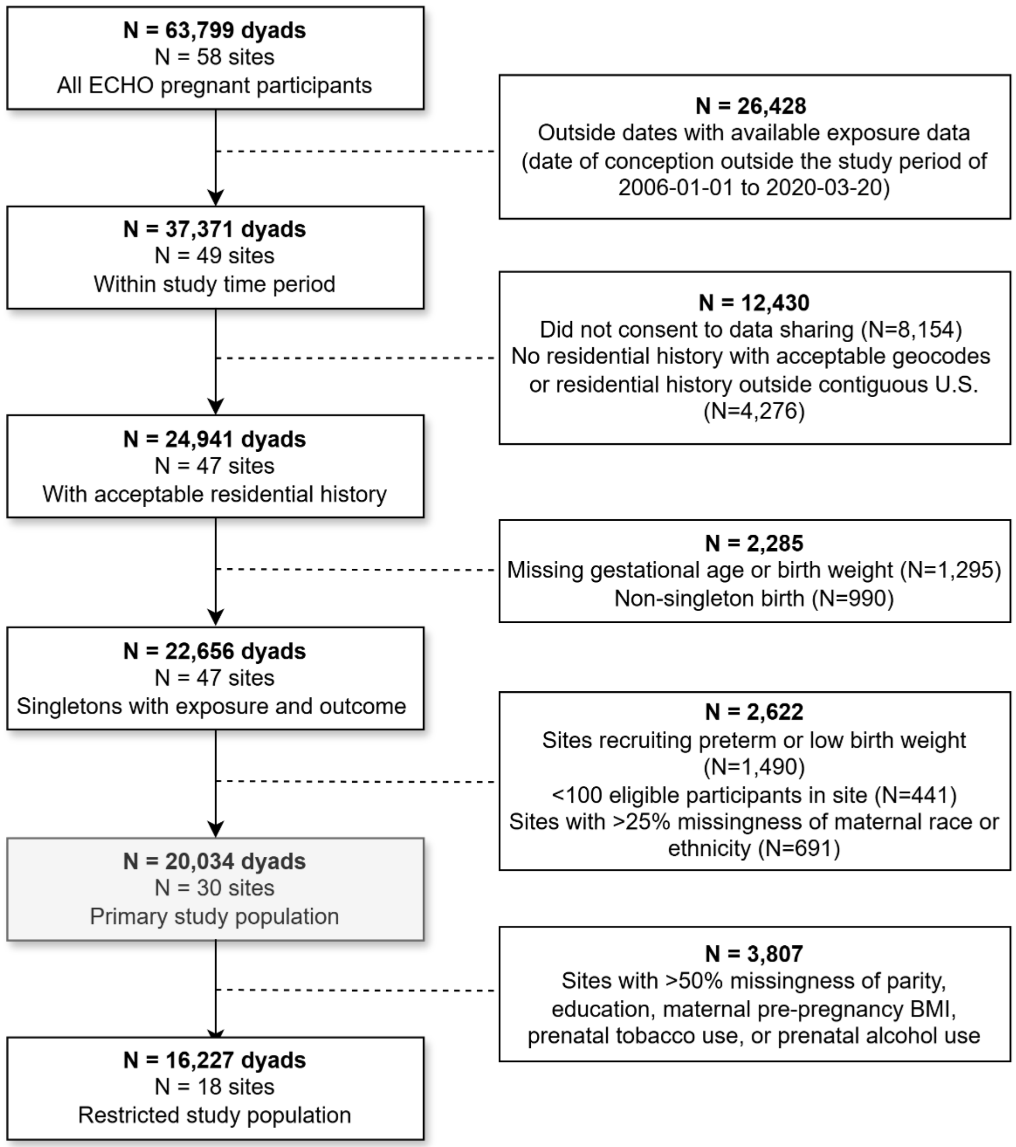


**Figure S2. Inclusion flowchart.**

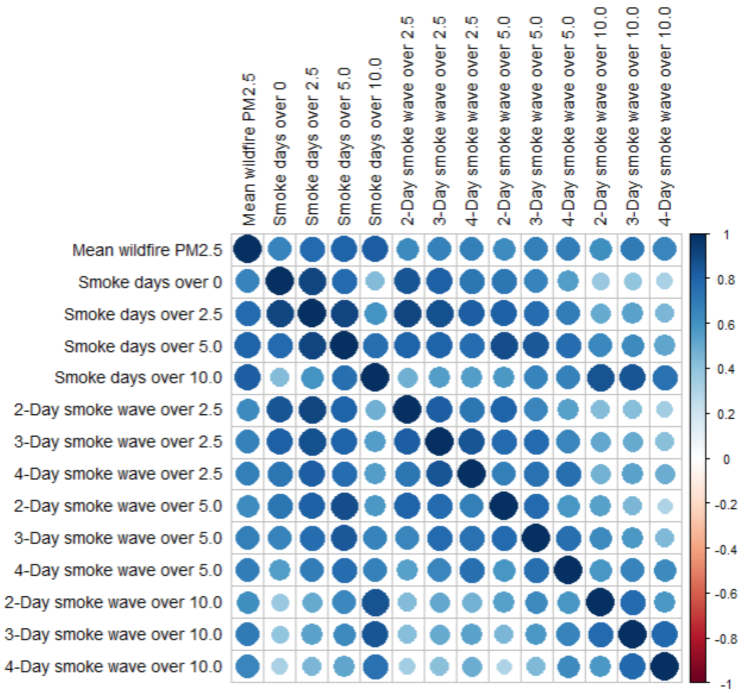


**Figure S3. Correlation between wildfire $PM_{2.5}$ exposure metrics during pregnancy.**

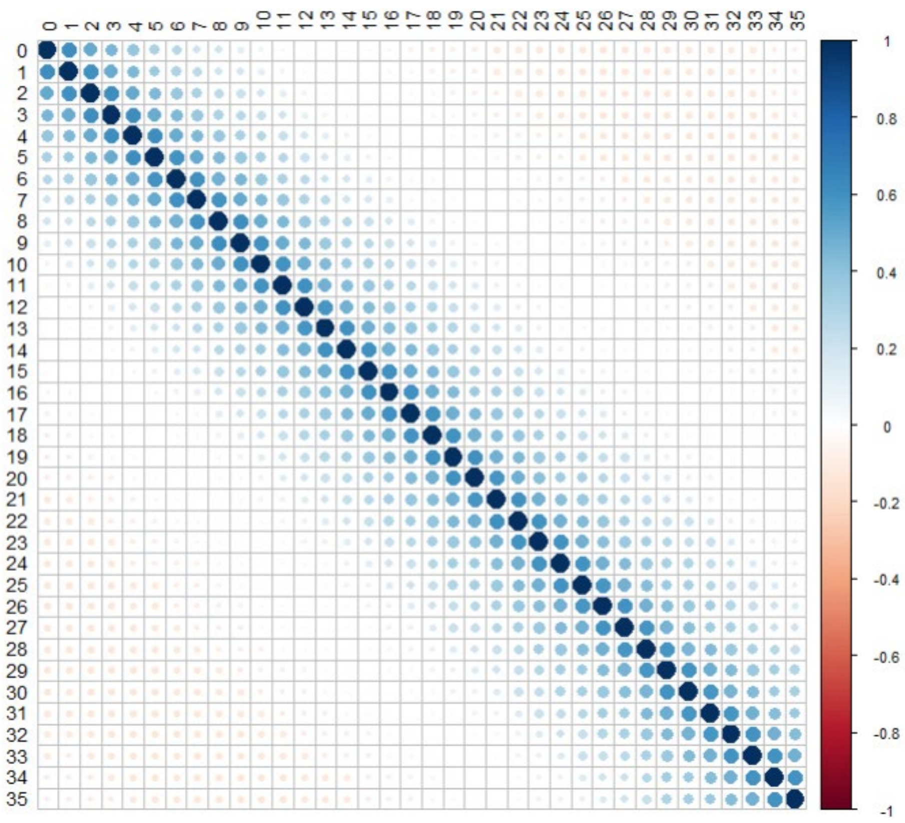


**Figure S4. Correlation between weekly smoke days across pregnancy.**

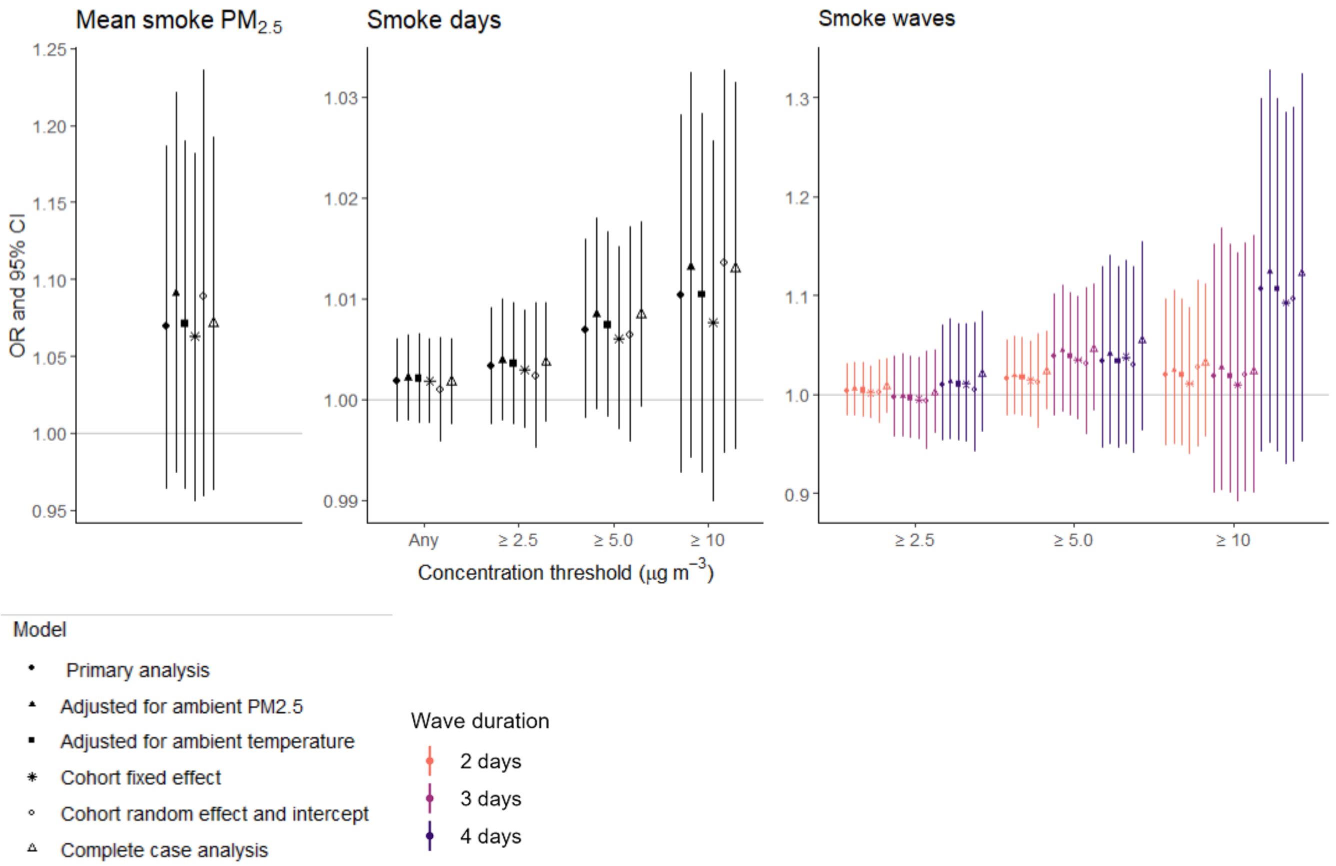


**Figure S5. Sensitivity analyses of the relationship between cumulative wildfire exposure and preterm birth in the nationwide study population.**

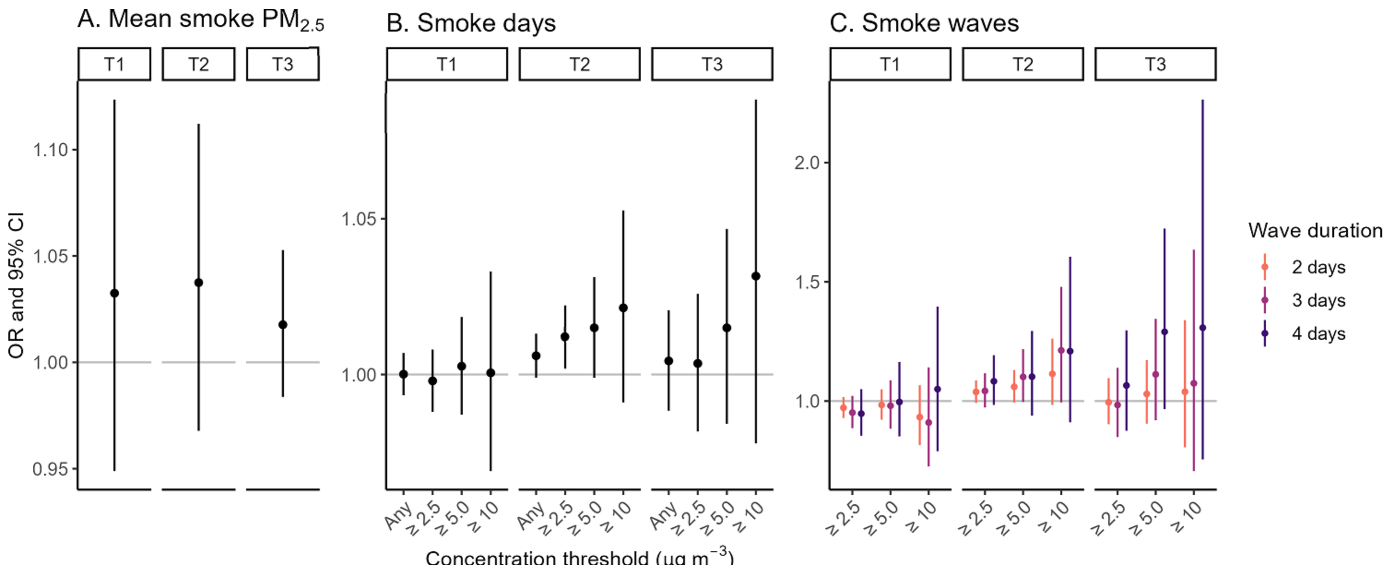


**Figure S6. Associations between trimester-specific exposure to mean wildfire $PM_{2.5}$, smoke days, and smoke waves during pregnancy and conditional odds of preterm birth in Trimester 1 (0-13 weeks), Trimester 2 (14-26 weeks), and Trimester 3. Trimester 3 was defined as the last 4 weeks before delivery and not evaluated for births <31 weeks gestation to avoid differential averaging period by gestational age. Associations determined by logistic regression adjusted for age, race, and ethnicity of the pregnant individual; child sex; neighborhood poverty rate; season of conception, year of birth splines (4 df), spatial splines (10 df), and cohort random intercept.**
